# Impact of thermal oxidation parameters on micro-hardness and hot corrosion of Ti-6Al-3Mo-2Nb-2Sn-2Zr-1.5Cr alloy

**DOI:** 10.1038/s41598-023-38216-4

**Published:** 2023-07-12

**Authors:** Fathy S. Ahmed, Mohamed A. El-Zomor, Magdy S. Abo Ghazala, Ramadan N. Elshaer

**Affiliations:** 1 Tabbin Institute for Metallurgical Studies, Cairo, Egypt; 2 Faculty of Science, Menofia University, Shebeen El-Koom, Egypt

**Keywords:** Engineering, Materials science

## Abstract

Protective oxide layers on Ti-6Al-3Mo-2Nb-2Sn-2Zr-1.5Cr (TC21) alloy with equiaxed microstructure considerably influence micro-hardness and hot corrosion resistance. The present work’s thermal oxidation of TC21 alloy was performed at 600, 700, and 800 °C for 5, 20, and 50 h durations. Hot corrosion methods in NaCl and NaCl + Na_2_SO_4_ salt media were applied to raw (unoxidized) and oxidized samples at 600 and 800 °C for 50 h. Hot corrosion was conducted at 600 °C for 5 cycles with 10-h steps. The best oxide layer thickness was observed at 800 °C, which increased with increased oxidation time and temperature. The surface hardness of the oxide layer at 800 °C was 900 ± 60 HV_0.05_ owing to the formation of TiO_2_ and Al_2_O_3_ phases. Raw material hardness was 342 ± 20 HV_0.05_, increasing threefold due to thermal oxidation. In the case of NaCl, weight loss dominated all samples except at 800 °C for 5 h. In the case of NaCl + Na_2_SO_4_, weight gain occurred at 600 and 800 °C for 5 h. Weight loss occurred for the raw samples and those processed at 800 °C for 20 and 50 h, where the oxide layer flaked off. Surface hardness increased upon hot corrosion testing because of the formation of brittle phases, such as TiO_2_ and Na_4_Ti_5_O_12_. Samples that oxidized at 800 °C for 5 h had the highest hardness and corrosion resistance.

## Introduction

Titanium is a low-density (60% the density of stainless steel) nonmagnetic alloy with outstanding thermal conductivity characteristics. Titanium alloys are widely used in many applications such as aviation, chemical industry^1–3^, petrochemical^4^, pharmaceutical^5,6^, biomedical industries, mining^7,8^, nuclear & energy generation, geothermal, desalination, heat exchangers^9^, etc. The properties of titanium alloys are affected by various factors, including alloying and microstructure^10–12^. These alloys have good properties such as strength, toughness, fatigue, corrosion, and thermal stability. However, a major factor affecting mechanical properties is microstructure. The important phases of titanium alloys are α-phase, hexagonal close-packed structure (HCP), and β-phase, body-centered cubic (BCC). α-β alloys are the most extensively used titanium alloys. Many microstructures of titanium alloys are formed, such as lamellar, equiaxed, and bimodal. The microstructure can be changed using different heat treatment regimes and cooling media. The microstructural parameters include morphology, grain size, volume fraction, and phase distribution^13,14^.

Generally, the equiaxed microstructure provides good strength, high ductility, and high fatigue resistance^15–17^. Mechanical characteristics are the most important performance factor for titanium applications such as movable and fixed blades in gas turbines. Biocompatibility is the most crucial element in medicine, such as dental implants and bone scaffolds. Corrosion is the most important property in the petroleum sector, such as fluid transmission tubes. So, the most crucial requirement in the industrial sector is corrosion resistance. Because titanium alloys are exposed to corrosion, oxygen can permeate deep through the metallic substrate to create an oxygen dissolution area, which causes the afflicted area to become more brittle^18–20^. Titanium alloys experience hot corrosion in the maritime environment and rapid oxidation over 400 °C in oxygen-containing settings^21^. In oxidizing acid environments and neutral chloride, titanium alloys demonstrate more resistance to corrosion than stainless steel. Due to a durable and protective oxide film whose nature is highly influenced by environmental variables; titanium is resistant to corrosion. When titanium is exposed to aqueous circumstances, the protective oxide instantly develops on the surface^1,22–24^. The oxide layers that form on the surface of titanium alloys continue to be resistant to both general and localized corrosion in the majority of oxidizing and neutral environments without the requirement for corrosion inhibitors such as ferrous and aluminum alloys, while they are exposed to reducing media^25,26^.

High strength, hardness, and toughness are characteristics of the TC21 Ti-alloy, which is regarded as a relatively novel form of α + β titanium alloy. TC21 alloy, which has the following chemical formula: has been effectively used by the aerospace industry. Building key components like landing gear connection boxes and airfoil joints with Ti-6Al-3Mo-2Sn-2Zr-2Nb-1.5Cr-0.1Si^13,16,17^. TC21 titanium alloys applied in the field are processed in salt-containing environments, such as boiler tubes and aerospace components. Sulfate and chloride salts may accumulate on the alloy surface^27^. In the case of aerospace engines, the components involving titanium alloys are exposed to temperatures in the range of 100–600 °C. Therefore, titanium alloy components undergo degradation (hot corrosion), decreasing engine lifetime. The significant factors influencing hot corrosion include alloy composition, deposit composition, deposit amount, temperature, and temperature cycles. The corrosion scales consisted of mixed Na–Ti–rich oxide and pores TiO_2,_ so the TiO_2_ layer was not protected, and spallation. Al_2_O_3_ affects hot corrosion resistance in chlorine-containing environments. Thus, the coatings for titanium alloys must be resistant to hot corrosion. Thermal oxidation protects alloys from hot corrosion. Based on titanium's natural attraction to oxygen and its diffusion at high temperatures, the thermal oxidation method is simple and relatively inexpensive^28–33^.

Titanium alloys are highly corrosion-resistant due to the formation of surface oxide layers at room temperature. The oxidation process involves the inward diffusion of oxygen in the titanium alloy, which forms a stable, adhesive, and protective layer mainly consisting of TiO_2_. The oxidation process becomes complex because of the presence of β-stabilizer alloying elements. The rutile TiO_2_ layer is unprotective and allows oxygen diffusion to the metal at high temperatures. The oxygen solubility in the α-phase is higher than in the β-phase. Oxide layer properties depend on composition and layer thickness, temperature, and oxidation time. The mechanical properties of oxide layers also change with the temperature of oxidation. Oxide layers are an effective corrosion barrier and enhance mechanical properties^34–36^.

Several coating techniques, including plasma electrolytic oxidation (PEO)^37^, plasma spraying^38^, and laser cladding^39,40^, are employed in an effort to improve the high-temperature oxidation resistance, hardness and wear resistance. The current area of study in corrosion and protection is the oxidation and corrosion behavior of titanium alloys^3,41–43^. For instance, Chen et al.^3^ investigate the corrosion degradation behavior of Ti6Al4V alloy in simulated marine environments. They found that this alloy demonstrated excellent corrosion resistance and only experienced a slight weight loss of 0.018 mg/cm^2^ throughout the 50 cycles salt spray because TiO_2_ passivation coating formed on the surface. According to Dai et al.^44^, Ti_2_AlNb alloy underwent a hot salt corrosion test, and the Cl_2_ product that resulted from that test was produced. This product then reacted with the substrate to create volatile chlorides, which caused the oxide layer to severely split. The oxidation behavior of the Ti6Al4V alloy between 500 and 600 °C was studied by Aca et al.^45^. They discovered that the primary atomic phases of the oxide layer were TiO_2_ and Al_2_O_3_. Fewer researchers, meanwhile, have studied on how hot corrosion affects an alloy's mechanical properties and how best to preserve it. The thermal oxidation approach was simple and inexpensive for creating a protective oxide layer. This study investigated the influences of the oxide layers formed by thermal oxidation at 600 and 800 °C for 50 h on the microhardness of TC21 Ti-alloy. In addition, the behaviors of raw and oxidized materials (O600 and O800 °C/50 h) upon NaCl and Na_2_SO_4_ hot corrosion at 600 °C were examined. Additionally, hot corrosion influences mechanical properties.

## Experimental procedure

TC21 Ti-alloy used in this investigation was received with φ 7 × 140 mm (Baoji Hanz Material Technology Co. Ltd., China) and composition 6.5Al–3Mo–1.9Nb–2.2Sn–2.2Zr–1.5Cr–0.09Si (wt. %). It had an equiaxed microstructure comprising the α (HCP) and the β (BCC) phases. The samples had a 7-mm diameter and 12-mm length. The samples were ground using SiC papers up to P800 grit and then ultrasonic-cleaned in ethanol. For the thermal oxidation process, the samples were heated in a compact vertical tube furnace (Carbolite EVC, United Kingdom). Samples were heated at 600, 700, and 800 °C for 5, 20, and 50 h, followed by air-cooling. The Mettler Toledo scale weighted the samples to an accuracy of 0.1 mg before and after oxidation. The oxidation sample determined weight change per unit surface area (mg/cm^2^) was used to compute activation energy.

In numerous stages, spraying utilized NaCl and a mixture of 75% Na_2_SO_4_ + 25% NaCl. This process continued until about 3.5 mg/cm^2^ of salts were added to the samples, and the surfaces were covered. The samples were heated in a muffle furnace at 600 °C for 5, 10, 20, 30, 40, and 50 h and then cooled in the air. The samples were cleaned in boiling distilled water and dried using hot air. Also, the samples were weighed before and after hot corrosion cycles. Consequently, samples were covered with salts to start the next corrosion cycle. Samples were produced using normal metallography procedures (cutting, mounting, grinding, and polishing), etched using Kroll's solution (3% HF, 30% HNO_3_, and 67% H_2_O), and then investigated by SEM. The morphology and cross-sections were analyzed by scanning electron microscopy (SEM, FEI INSPECT 50S, The Czech Republic) and energy-dispersive spectroscopy (EDS, Bruker AXS-Flash Detector 410-M, Germany). Image J software measured the oxide layer thickness and the corrosion scale. LECO LM700 microhardness tester measured the hardness of the oxide layer and the corrosion scale with a load of 50 gf for a duration of 15 s. The hardness is measured as follows the first point was taken through surface morphology samples and other measurements were taken cross section samples. The average hardness was applied by five indentations to each sample. The oxidation and corrosion phases were analyzed by X-ray diffraction (PANalytical X’Pert PRO, The Netherlands) with source a monochromatic CuKα and 2θ ranging from 20° to 80°.

## Results and discussion

### Microstructure evaluation

**Figure 1:**
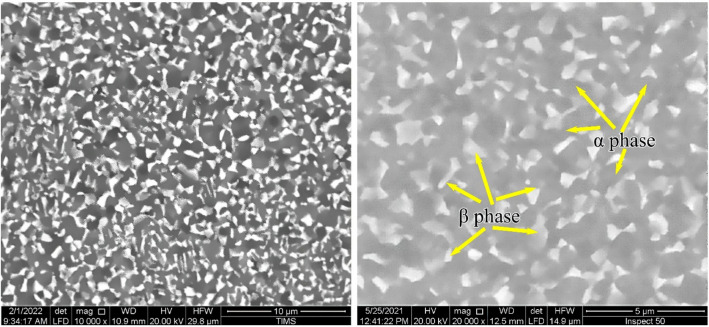
Equiaxed microstructure consists of primary α and β-phases.

Figure 1 shows the equiaxed microstructure of the substrate. The equiaxed microstructure consisted of the primary α-phase with a 65% volume fraction and the regularly distributed β-phase with a grain size of 2.5 μm. The volume fraction had a high percentage due to the slow cooling rate, allowing for equiaxed α-grains to grow. The α and β-phases were EDS analyzed and represented in Table 1. Al (α-stabilizing element) had a concentration of 15.2 at.% in the α-phase but only 10 at.% in the β-phase. Mo, Nb, and Cr (β-stabilizing elements) had concentrations of 1.2, 0.3, and 1.8 at.%, respectively, in the β-phase. Neutral elements such as Zr and Sn existed in the α and β-phases with approximately the same amount.

**Table 1:** EDS analysis of α and β phases, at.%.

Samples	Point	Al	Ti	Cr	Zr	Nb	Mo	Sn
Raw	α	15.2	81.1	1.1	0.2	0.1	0.9	1.4
	β	10	85.4	1.8	0.1	0.3	1.2	1.2

### Thermal oxidation

**Figure 2:**
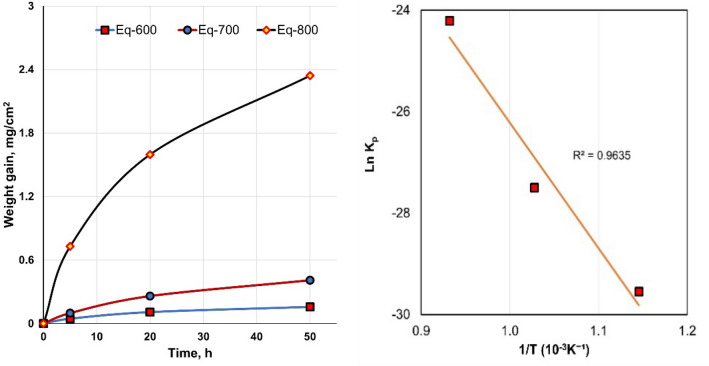
Thermal oxidation weight gain of equiaxed microstructure samples related to oxidation time at temperatures 600, 700, and 800 °C for 50 h. Arrhenius equation representation of parabolic constant (log K_p_) with temperature (1/T) at the same conditions.

Figure 2 shows the weight gain per surface area over time at different temperatures. The thermal oxidation process was applied in the air at 600, 700, and 800 °C for 50 h every 10 h, where the oxidation rate had a parabolic behavior. The parabolic rate constant K_p_ was associated with forming a brittle and hard oxide layer for the given temperature range. The parabolic rate constant K_p_ increased with the oxidation temperature. The value of K_p_ increased nearly eightfold as the oxidation temperature increased from 600 to 700 ℃, whereas K_p_ highly increased twenty-sixfold as the oxidation temperature increased from 700 to 800 ℃. Furthermore, the Arrhenius equation was used to show the relation between Ln K_p_ against 10^3^/T, where the obtained linear plot and the slope can determine the activation energy for oxidation. The activation energy differed for the same alloy, depending on the oxidation temperature and time^46^.

The Arrhenius equation was used to determine activation energy from the thermal oxidation method by relating K_p_ to T (temperature)^14^.

1 $${\text{K}}_{{\text{p}}} = {\text{K}}_{{\text{o}}} {\text{exp}}^{{ - ({\text{Q}}/{\text{RT}})}}$$

where K_o_ represents the pre-exponential factor, Q is the activation energy, R (8.3143 J/(mol K)) is the gas constant, and T (°K) is the temperature. The activation energy (Q_ox_) for the oxidation of equiaxed TC21 alloy (for 50 h) was determined as 232 kJ mol^−1^. The activation energies of Ti-6Al-4 V alloy at oxidation between 600 and 700 °C were 276 and 191 kJ mol^−1^, respectively, for 72 h. The activation energies of commercially pure (CP) Ti alloy at oxidation temperatures between 500 and 800 °C were 275 kJ mol^−1^ for 72 h. The activation energy for Ti–6Al–7Nb was 170 kJ mol^−1^^19,47,48^.

**Figure 3:**
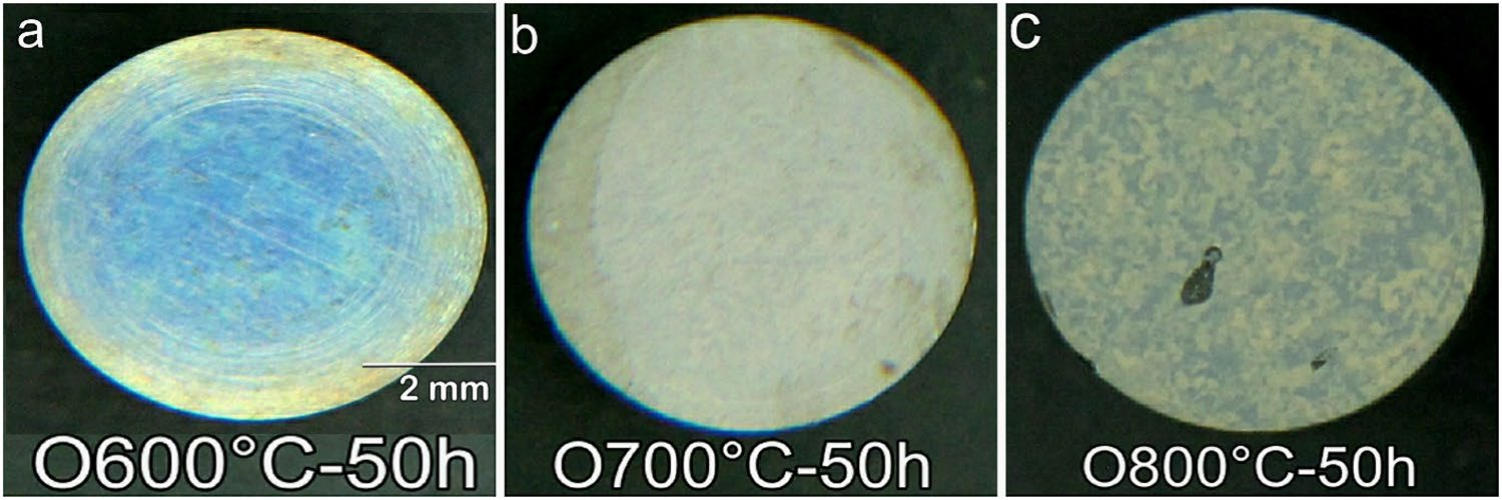
The color appearance of the oxidized samples under different oxidation temperatures for 50 h: (**a**) 600 °C, (**b**) 700 °C, (**c**) 800 °C.

The sample weight gain was 0.16 mg/cm^2^ for the sample oxidized at 600 °C for 50 h (O600-50). Increasing the oxidation temperature to 700 °C for 50 h (O700-50) increased the weight gain to 0.41 mg/cm^2^. The high oxidation temperature at 800 °C for 50 h (O800-50) led to a 2.3 mg/cm^2^ weight gain, sixfold higher than that of O700-50. The colors of the oxidized samples changed with temperature, Fig. 3. The blue-to-clay group was observed from 600 to 800 °C for 50 h. The thickness and composition of the oxide layer changed with the increased oxidation temperature, which led to the associated change in the interference of the incoming light. No spallation and cracks were observed on O600-50 and O700-50, but O800-50 involved small spallation (bark region).

**Figure 4:**
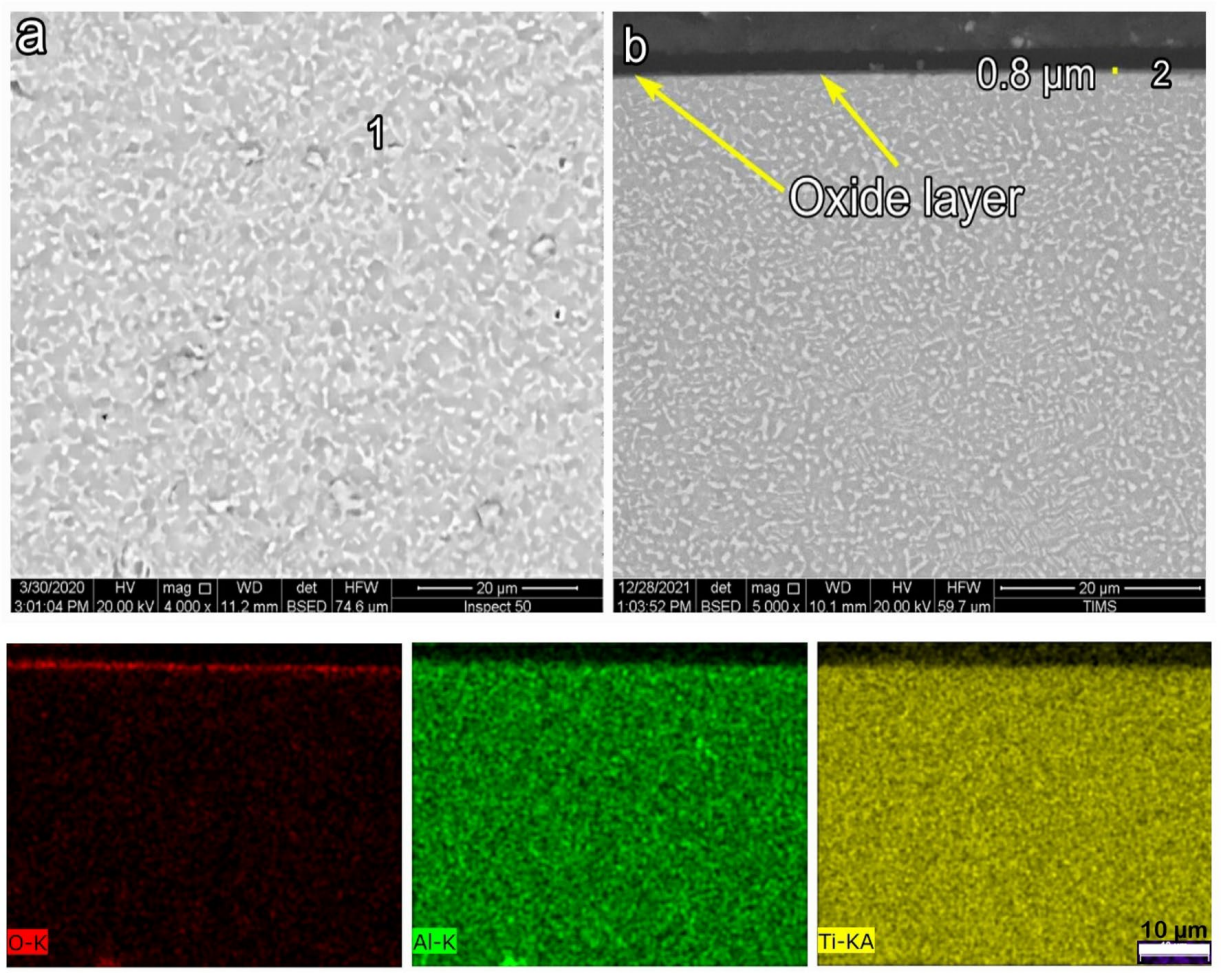
SEM and EDS analysis for samples O600-50 (**a**) morphology, (**b**) cross-section, and (**c**) element map concentration.

SEM images presenting the morphology and cross-section of O600-50 are shown in Fig. 4. SEM morphology showed an equiaxed microstructure after oxidation, where oxide grains did not appear, as shown in Fig. 4a. The EDS point analysis showed that the morphology of the oxide layer was mainly composed of Ti, O, Al, and a tiny amount of another alloying element (point 1), as presented in Table 1. The back-scattered SEM image showed a very thin, continuous, and homogeneous oxide layer for O600-50, as shown in Fig. 4b. The oxide layer thickness increased from 0.23 to 0.8 μm as the time increased from 5 to 50 h, respectively. The Al elemental concentration maps were not present in the cross-section of the oxide layer. The Al concentration at point 1 and point 2 was 4.9 and 1.8 at%, where this value reduces to Al concentration in base metal. Based on EDS elemental mapping, it was determined that the titanium oxide layer formed very thinly, which was further validated by the analysis shown in Table 2. Figure 8 shows that the oxide layer’s thickness increased with the oxidation time, where the oxide layer increased threefold as the time increased from 5 to 50 h^49^.

**Table 2:** EDS point analysis (at.%) for oxidation morphology and cross-section samples as follows.

Samples	Point	O	Al	Ti	Cr	Zr	Nb	Mo	Sn
O600-50	1	55.7	4.9	37.4	0.7	0.3	0.4	0.4	0.2
	2	69	1.8	29.2	–	–	–	–	–
O700-50	1	61.1	3.7	34.5	0.4	–	–	0.2	0.1
	2	66.4	3.4	30.2	–	–	–	–	–
O800-5	1	64.8	6.3	28.4	0.2	0.2	–	–	0.1
	2	65.6	5.8	28.6	–	–	–	–	–
O800-50	1	64.2	6.4	29.1	–	0.1	0.1	–	0.1
	2	68.4	4.7	26.9	–	–	–	–	–

**Figure 5:**
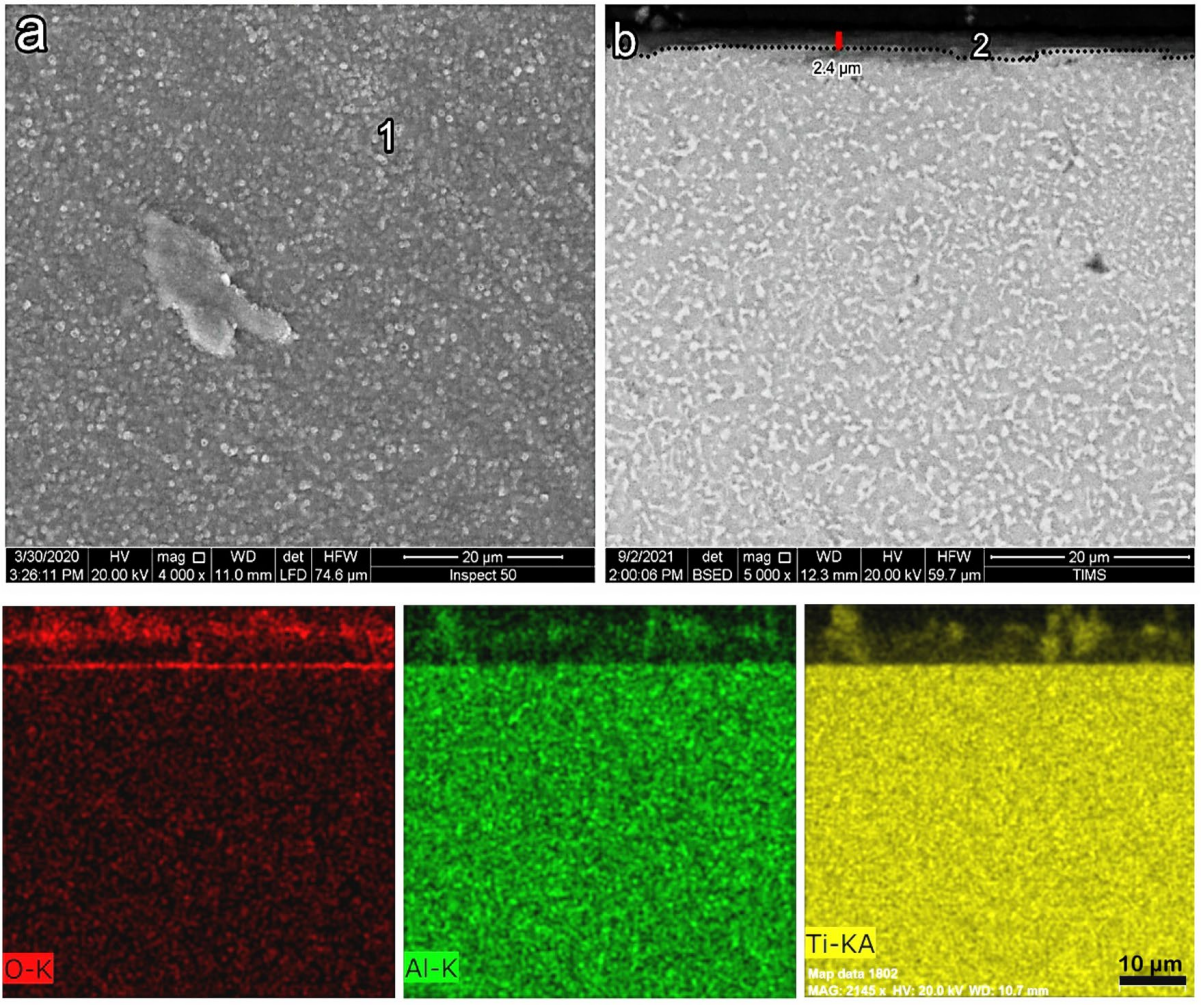
SEM and EDS analysis for samples O700-50 (**a**) morphology, (**b**) cross-section, and (**c**) element map concentration.

Figure 5 shows the morphology and cross-section of O700-50. Oxide grains were tiny, and an equiaxed microstructure morphology appeared, as shown in Fig. 5a. The oxide layer thickness was measured as 2.4 μm, and the layer was continuous and homogeneous, as shown in Fig. 5. The EDS elemental maps for Al were not presented in the cross-section of the oxide layer, confirmed by point 1 EDS analysis. Point 2 in the oxide layer had the same concentration as O600 (point 2), suggesting that the same phase was formed. Figure 8 shows the effects of the oxidation temperature and time on the thickness of the oxide layer. The thickness of O700-50 was threefold higher than that of O600-50. The oxidation time highly affected the oxide layer thickness, which increased fourfold between 5 and 50 h.

**Figure 6:**
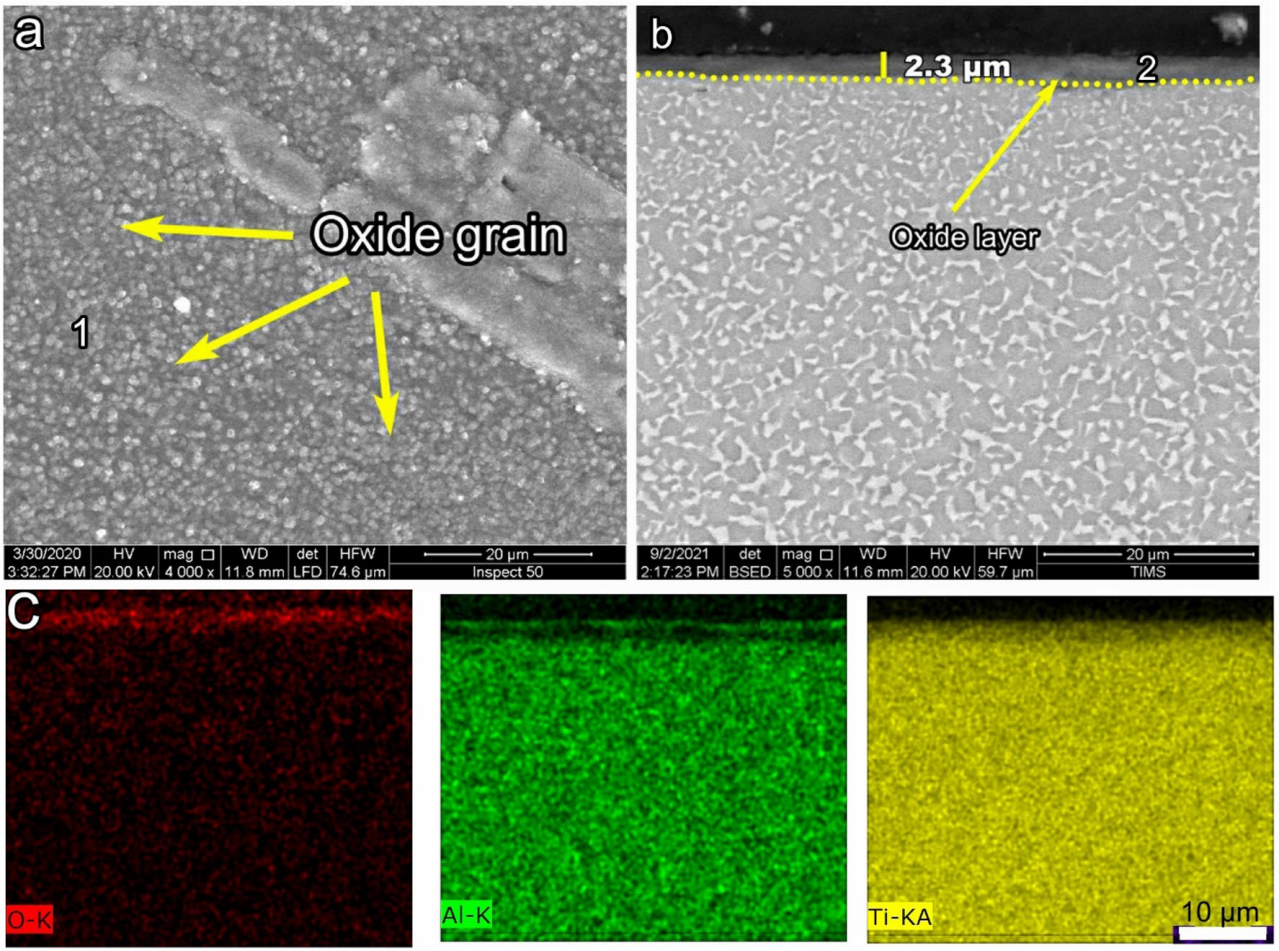
SEM and EDS analysis for samples O800-5 (**a**) morphology, (**b**) cross-section, and (**c**) element map concentration.

SEM and EDS images showing the morphology and cross-section of O800-5 are shown in Fig. 6. In the investigation, O800-5 was added after O800-50 failed to provide good corrosion properties. The oxide grain size increased with temperature and time because of the nucleation and aggregation of finer oxide grains^50^. The oxide grain morphology of O800-5 was coarser than that of O700-50, as shown in Fig. 6a. The back-scattered SEM image showed a continuous and homogeneous layer; no spallation was found for O800-5. The oxide layer thickness of O800-5 was 2.3 μm, the same thickness found for O700-50, as shown in Fig. 6b. The Al elemental maps presented thin and continuous layers on the upper oxide layers, Fig. 6c. The oxide layer morphology of O800-5 was analyzed by EDS, where point 1 comprised Ti, O, and Al, the same as that of point 1 obtained for O800-50, as presented in Table 2. The cross-section of O800-5 was composed of Ti, O, and Al, represented at point 2, and this result conformed to point 2 of O800-50, as shown in Table 2. The EDS point analyses for O600-50, O700-50, O800-5, and O800-50 were nearly the same, indicating stoichiometric TiO_2_ and a tiny Al_2_O_3_ phase detected in O800-5 and O800-50.

**Figure 7:**
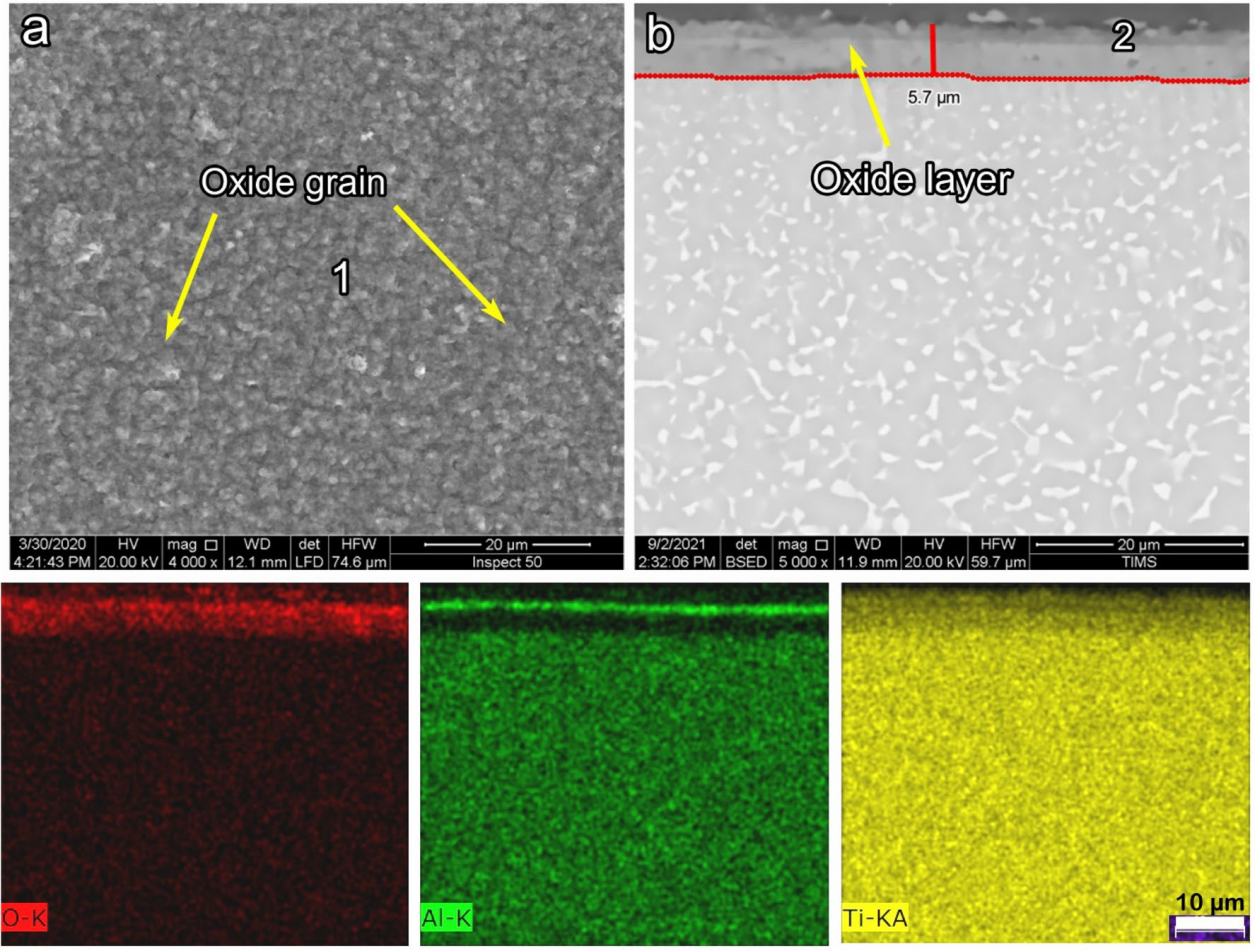
SEM and EDS analysis for samples O800-50 (**a**) morphology, (**b**) cross-section, and (**c**) element map concentration.

Figure 7 shows the SEM and EDS images for the morphology and cross-section of O800-50. Due to the nucleation and aggregation of oxide grains than O800-5, the oxide grain size increased over time. The oxide layer thicknesses of O800 samples were approximately proportional to time. Figure 7b shows that the average oxide layer was 5.7 µm, where the thickness of O800-50 was nearly twice that of O800-5. Observed in the BSE cross-section image, the oxide layer structure consists of two different layers, where the top layer seems enriched in Al, as shown in the element map. This is the sample where a continuous Al_2_O_3_ film is formed.

**Figure 8:**
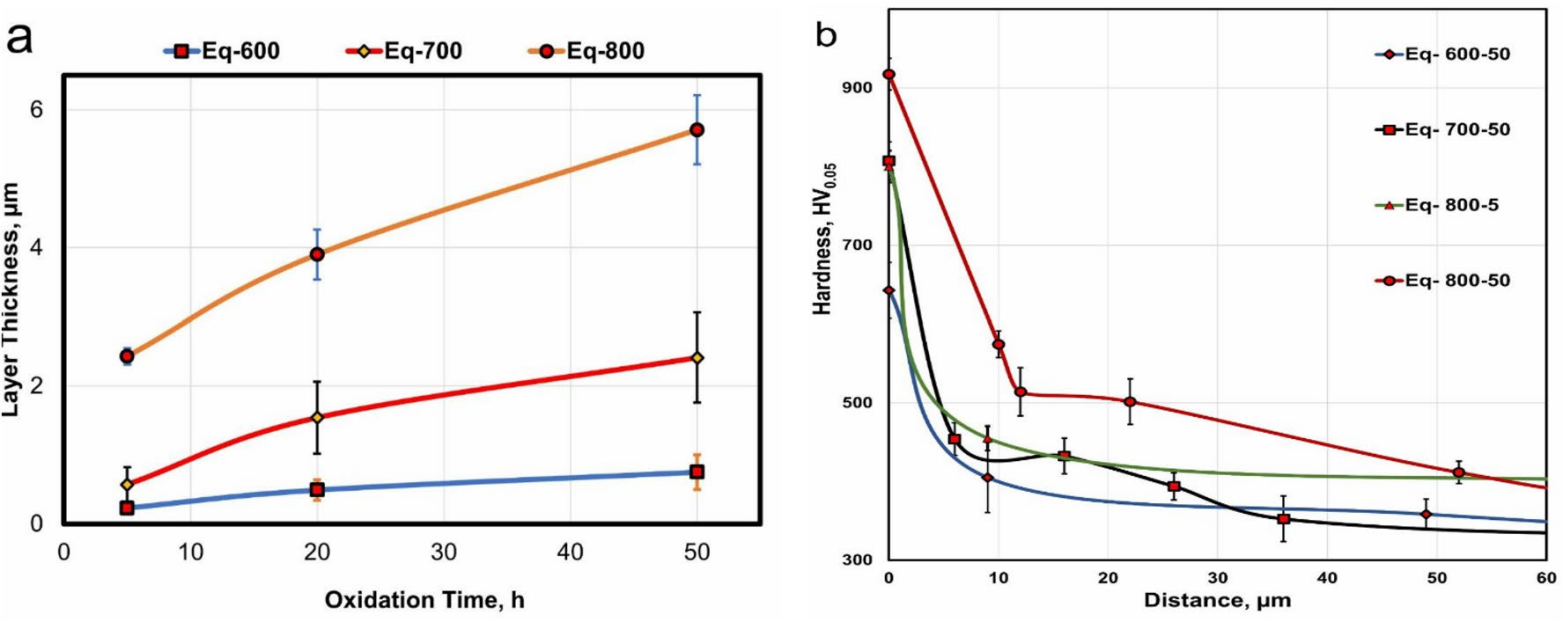
Thermal oxidation temperature related to (**a**) thickness of oxide layer, (**b**) hardness.

Figure 8a shows the average layer thickness as a function of oxidation time at different temperatures. The thickness oxide layer depended on oxidation temperature, where the temperature increases from 600, 700, and 800 ℃ led to an increase in oxide layer thickness by two- to seven-fold. According to the literature, the interstitial diffusion of oxygen in titanium alloys significantly increases the hardness at elevated oxidation temperatures, where oxygen diffusion increases, thereby increasing hardness^51^. The hardness of an oxide layer is proportional to the oxidation temperature and duration^52^. Figure 8b represents the microhardness measurements related to sample depths at different oxidation temperatures for 50 h. The values of the hardness of O600-50, O700-50, O800-5, and O800-50 show steady values from a depth of approximately 20, 36, 120, and 170 μm to the center of the specimen. The oxygen diffusion zone in the region below the oxide layer increases in thickness with increasing oxidation temperature and is responsible for the high hardness of this region. The hardness of O600-50 and O700-50 were 642 ± 36 and 807 ± 63 HV_0.05_, demonstrating that their hardness increased one-and-half-fold compared with those of raw samples (342 ± 20 HV_0.05_). O800-5 and O800-50 were 800 ± 20 and 900 ± 60 HV0.05, with the latter having the highest hardness. As a result, the oxide layer thickness increased and formed a hard oxide.

**Figure 9:**
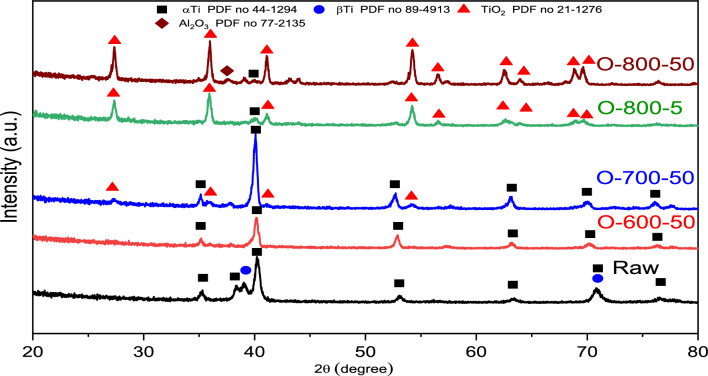
XRD raw and thermal oxidation patterns for samples at different temperatures. *Reference codes for each detected phase: α-Ti 44-1294, β-Ti 89-4913, TiO_2_ 21-1276, and Al_2_O_3_ 77-2135.

Figure 9 represents the XRD patterns of raw and oxidized samples at different temperatures. The raw sample was composed mostly of α-Ti and β-Ti phases, representing α-Ti as the dominant phase. O600-50 samples were mainly composed of α-Ti and a minor TiO_2_ phase. Since O is an α-phase stabilizer, it formed a thin α case under the oxide layer. The α-Ti phase intensity was very small in O800-5 and O800-50. The rutile TiO_2_ was noticeably small in O700-50 but became more intense in O800-5. So, the oxidation temperature affected the formation and stability of the phases. Al_2_O_3_ phases were present in O800-50, where Al_2_O_3_ formed a very thin layer, but the oxide layer was not continuous due to spallation.

Ti has a high affinity to O and thus forms a very thin oxide layer at room temperature. As Ti is heated in the air, the oxide layer increases with the heating temperature and time. As a result, the fast inward diffusion of O^2−^ and the fast outward diffusion of Ti^4+^ form pores and cracks and cause spallation. The results showed that O600 samples formed continuous, adherent, homogeneous, but very thin oxide layers. The increased oxidation temperature (O800) resulted in enhanced oxide layer thickness and good mechanical properties, but the oxide layer was spalled at extended oxidation times. So, the O600 and O800 samples were selected to study hot corrosion.

### NaCl hot corrosion

**Figure 10:**
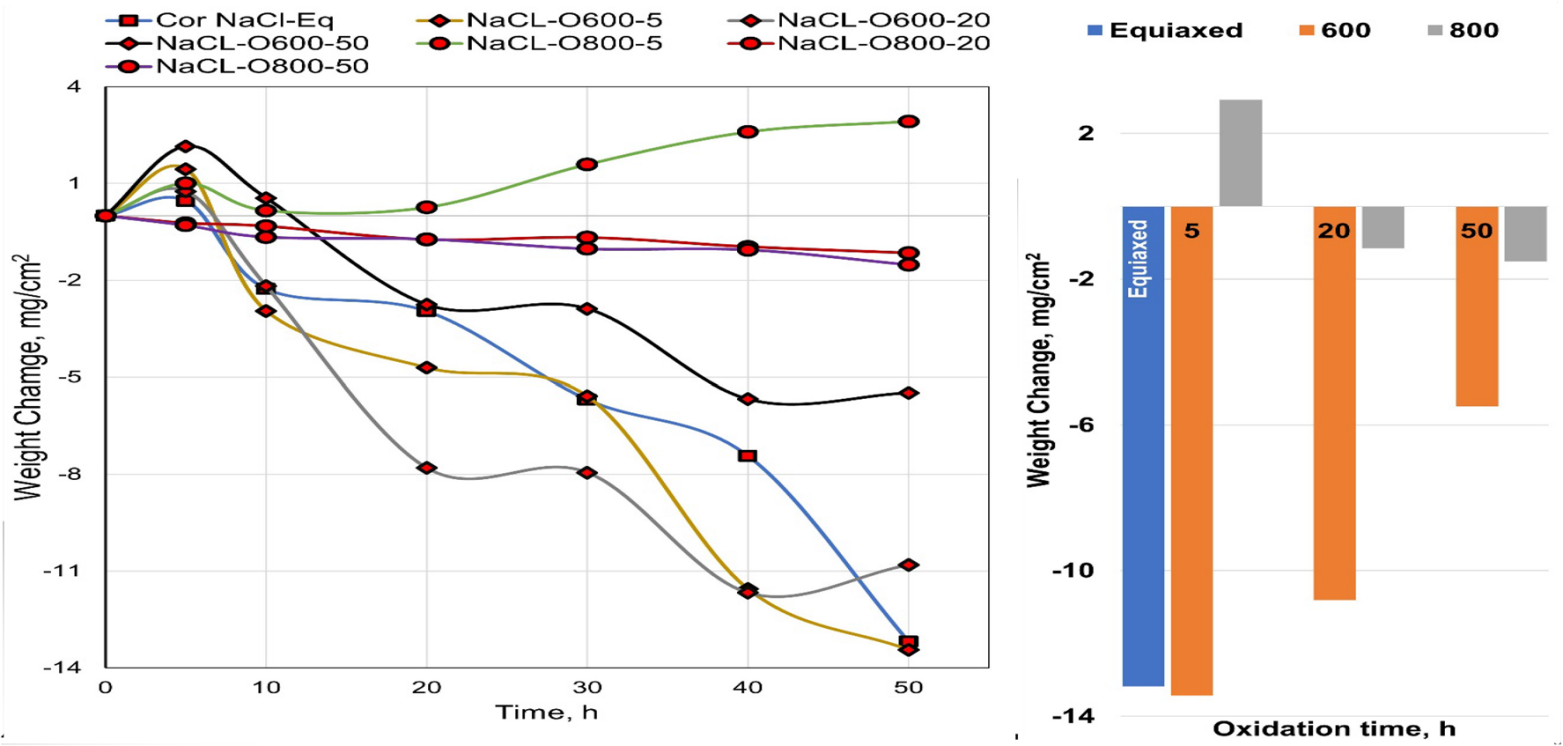
Weight change as a function of NaCl hot corrosion time for raw O600 and O800 samples at 600 °C.

**Figure 11:**
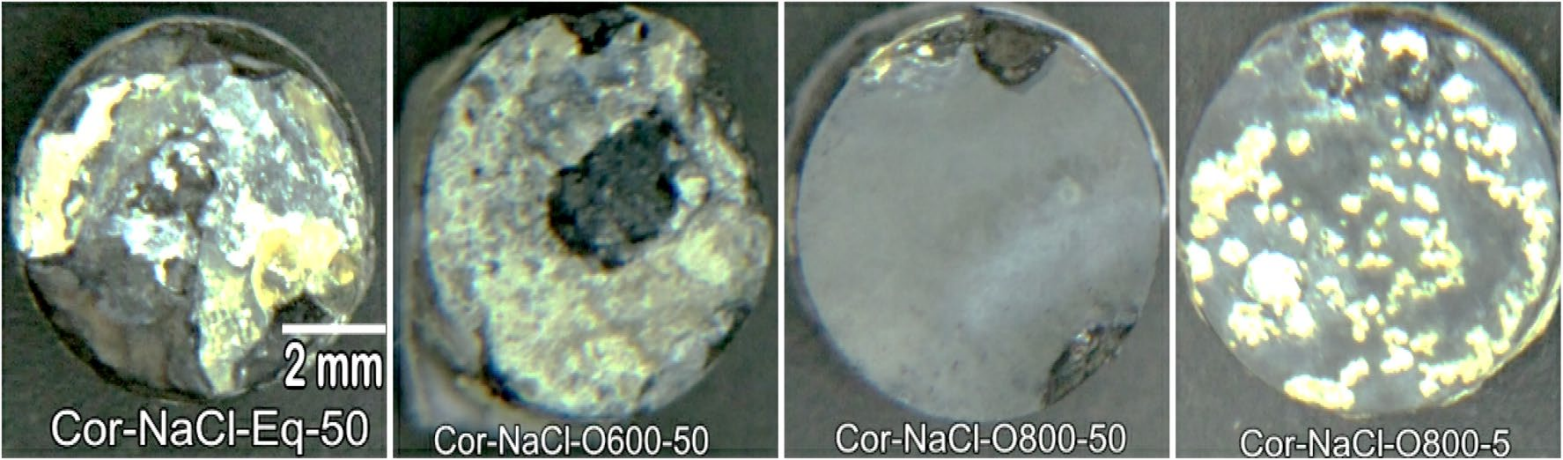
Surface morphology of raw, O600-50, O800-5, and O800-50 under NaCl hot corrosion at 600 °C for 50 h.

The presence of solid NaCl hot corrosion deposits on the alloy surface causes catastrophic damage. Figure 10 presents the weight change of raw, O600, and O800 samples after the NaCl hot corrosion cycle at 600 °C with 10-h steps for 50 h are shown in line and bar charts. Figure 11 shows the surface morphology of raw and oxidized samples after NaCl hot corrosion, representing a change in color and deformation in shape. For raw specimens, the metallic-white color was changed to black and yellow after corrosion, as shown in Fig. 11. The weight after 5 h was increased and then dropped between 5 and 10 h, and the weight loss between 10 and 50 h was relatively linear. The weight loss of raw samples was 13.2 mg/cm^2^ after hot corrosion because of corrosion scale spalling. Raw and O600-50 flaked off, and the samples showed high surface roughness with an irregular outer shape. In the case of O600, the specimen color changed to black after corrosion due to spallation and yellow as salt, as shown in Fig. 11. Initially, the weight after one cycle increased; then, the weight dropped after the second cycle. The weight losses for O600-5 and O600-20 were higher than that for O600-50, where nearly the same weight loss of raw samples was less protective because of the lack of oxide layers. The weight loss of O600-50 was 5.5 mg/cm^2^, as shown in Fig. 10. The appearance of O800 samples changed after corrosion, where the dark point appeared near the edge as spallation and the yellow spot due to the salt, as shown in Fig. 11. The weight gain of O800-5 was 2.9 mg/cm^2^ after all corrosion cycles. The weight losses of O800-20 and O800-50 were slightly influenced after all corrosion cycles, where the oxide layer caused spallation. The weight loss at O800-50 was 1.5 mg/ cm^2^, relatively low compared with raw and O600 after corrosion cycles.

**Figure 12:**
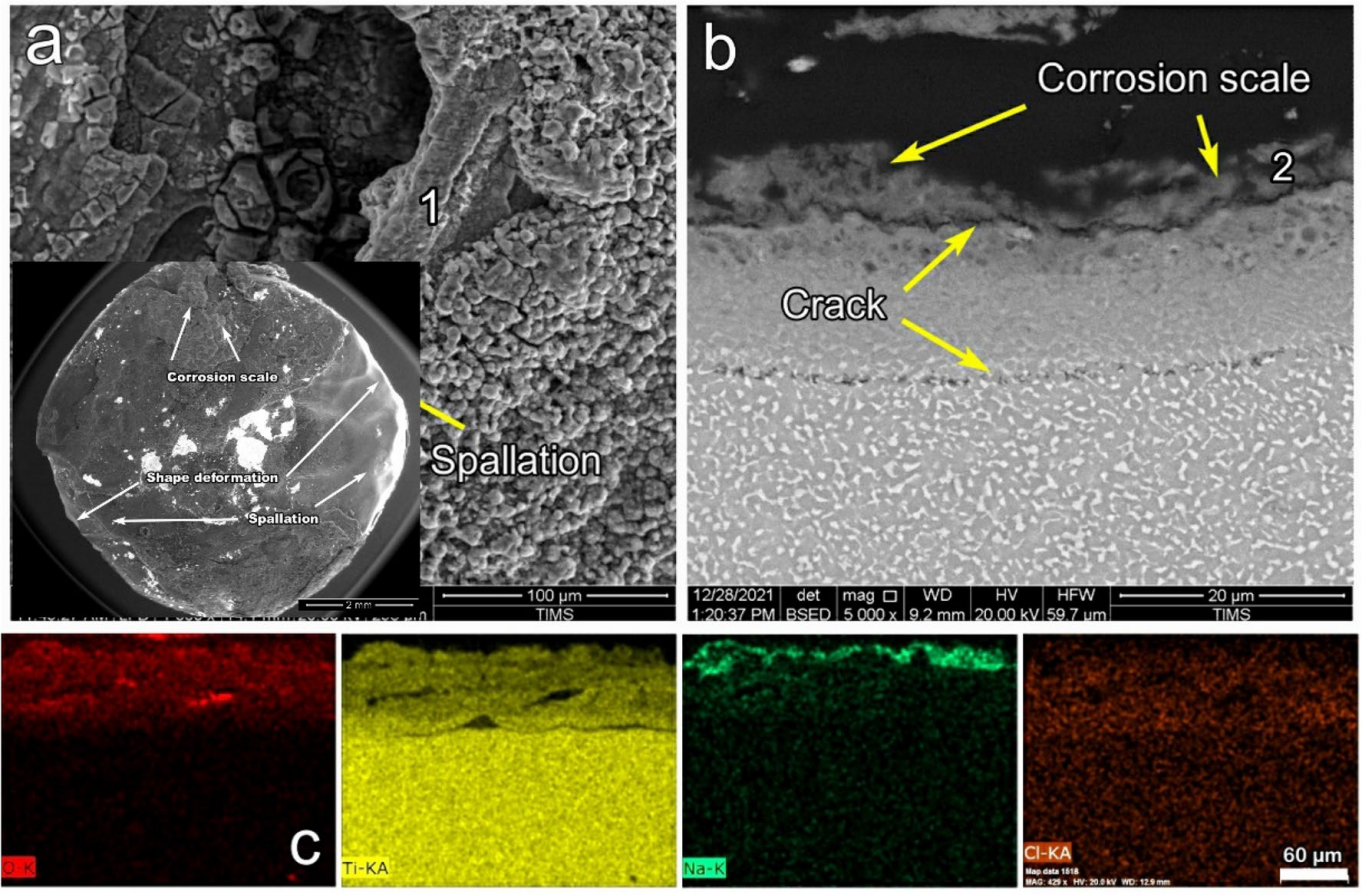
SEM and EDS element map concentration of raw samples after NaCl hot corrosion at 600 °C during 5 cycles.

Figure 12 shows SEM and EDS elemental maps of raw samples after NaCl hot corrosion (raw-NaCl) at 600 °C for 5 cycles. The morphology of raw samples included holes, blisters, and high porosity, as illustrated in Fig. 12a. The average thickness of the produced corrosion scales was 13 μm, indicating poor adhesion between the base metal. Some cracking was observed at a depth of 20 μm in the substrate parallel to the corrosion scale. The EDS elemental map represented O, Ti, Cl, and Na throughout the corrosion scale, as seen in Fig. 12c. Na was observed at the top surface of the scale at EDS point 1, where the average thickness of the Na layer was 18 µm. The O was presented with a depth penetration of 80 µm from the surface. Cl was also presented on the surface of the corrosion scale at point 1, as shown in Table 3. The cross-sections of raw-NaCl samples were composed of Ti, O, Na, and Al, represented at point 2, as shown in Table 3.

**Table 3:** EDS point analysis (at.%) for raw and O600-50 and O800-50 morphology and cress section samples after NaCl hot corrosion at 600 °C.

Samples	Point	O	Na	Al	Cl	Ti
Raw -NaCl	1	56.6	9.9	3.6	0.3	29.6
	2	64.6	8.2	2.7	–	24.5
O600-50-NaCl	1	68.4	7.8	0.6	0.4	22.8
	2	67.1	9.6	0.5	–	22.8
O800-50-NaCl	1	58.2	0.3	7.5	0.6	33.4
	2	61.2	0.1	6.4	–	32.3

**Figure 13:**
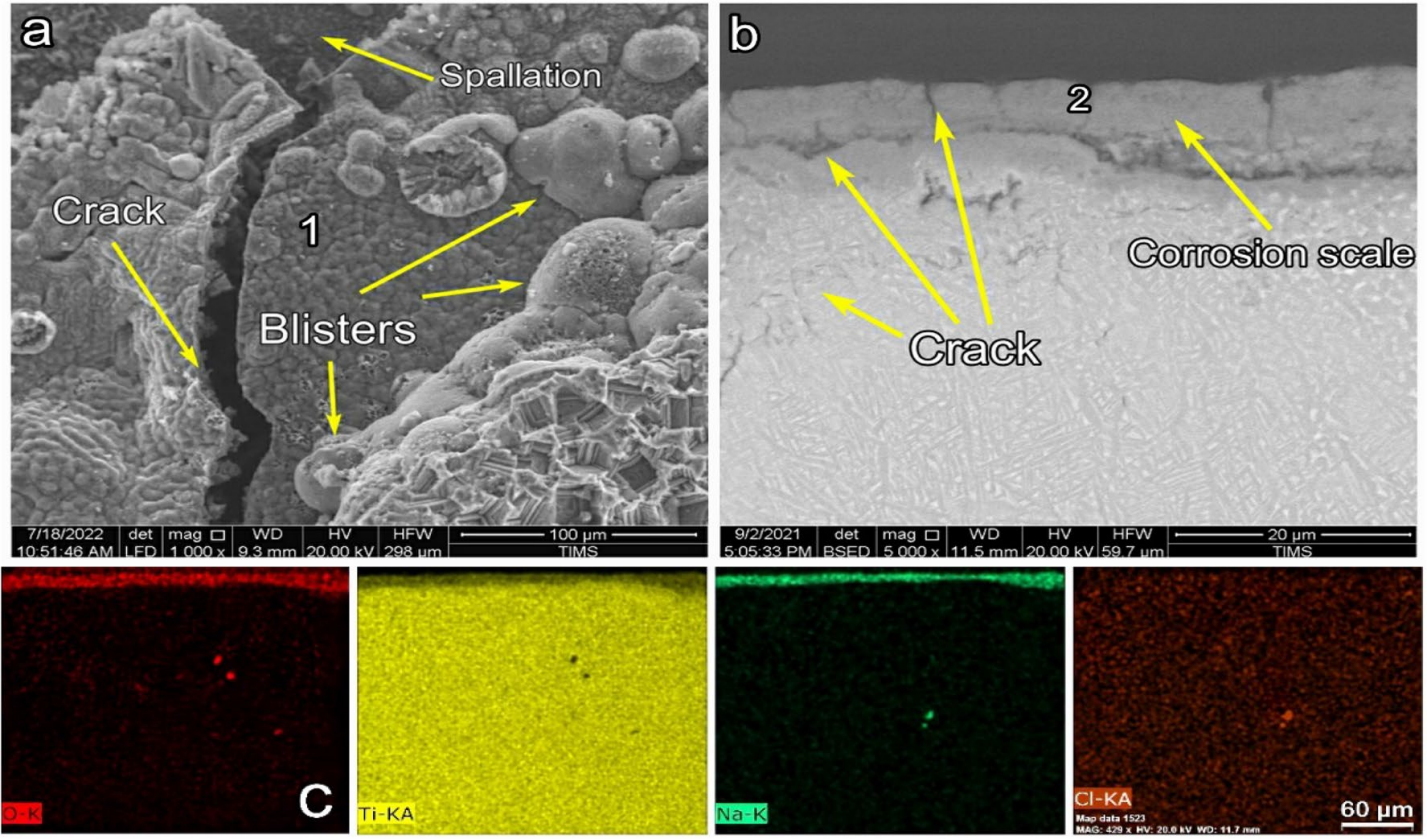
SEM and EDS element map concentration of O600-50 micrograph after NaCl hot corrosion at 600 °C for 5 cycles.

Figure 13 shows SEM and EDS elemental maps of O600-50 samples after NaCl hot corrosion (O600-50-NaCl) at 600 °C for 5 cycles. The SEM morphology of O600-50 samples comprised cracks, many blisters, and spallation, as illustrated in Fig. 13a. SEM cross-section represented poor adhesion and cracks, as shown in Fig. 13b. The average thickness of the scale was 15 µm. The Na layer formed on the upper surface had a 6 µm thickness. O was presented in the hole of the corrosion scale, while Cl was not presented. The morphology of O600-50-NaCl was analyzed by EDS, where point 1 was composed of Ti, O, Na, Cl, and Al, as presented in Table 3. The cross-section of O600-50-NaCl was analyzed at point 2, which agreed with point 2 in raw-NaCl, as shown in Table 3. The EDS point analyses of raw-NaCl and O600-50-NaCl showed high Na concentration on the corrosion scale and reflected stoichiometric sodium titanium oxide.

**Figure 14:**
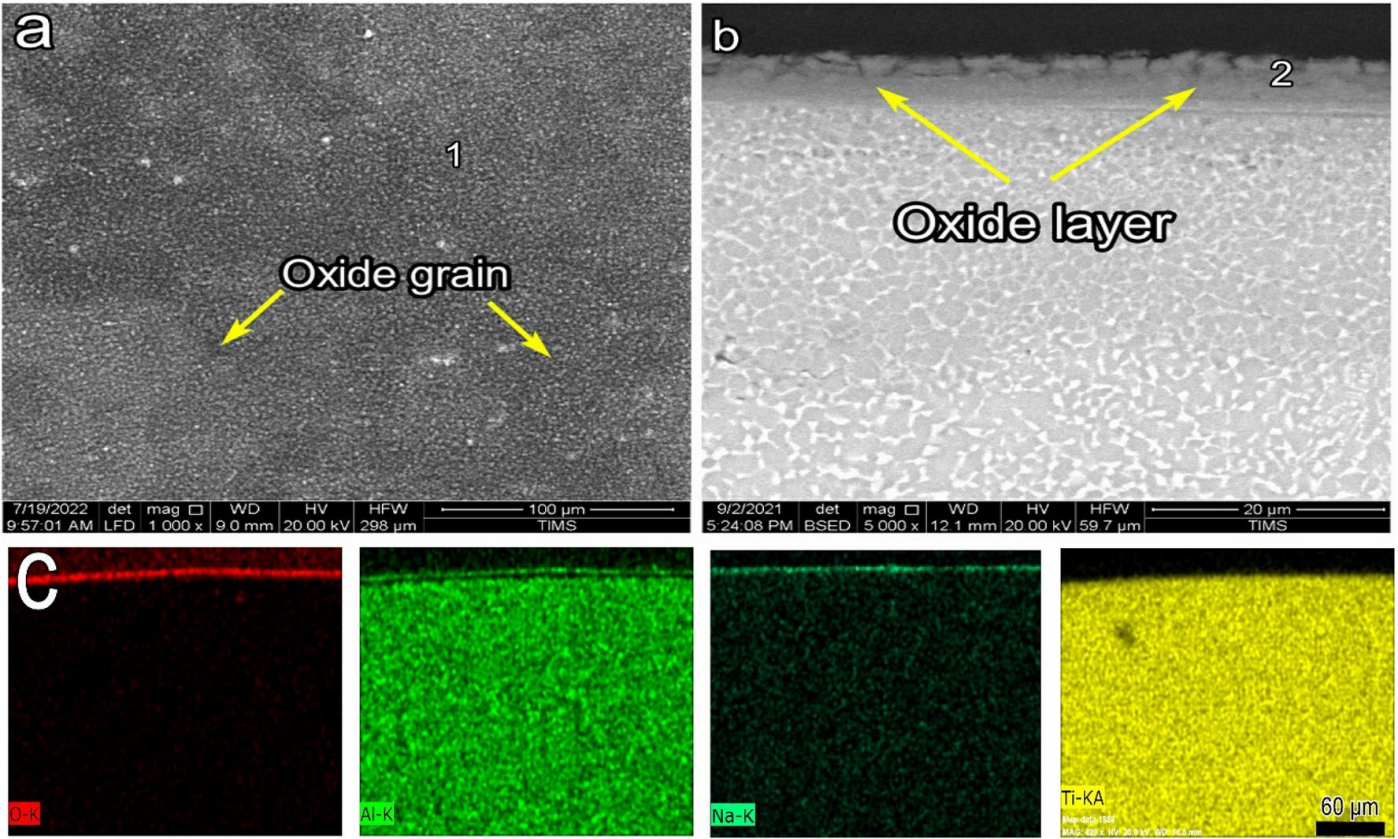
SEM and EDS element map concentration of O800-50 samples after NaCl hot corrosion at 600 °C during 5 cycles.

Figure 14 shows SEM and EDS elemental maps of O800-50 samples after NaCl hot corrosion (O800-50-NaCl) at 600 °C for 5 cycles. The resistance of corrosion increased with temperature (O800). The SEM morphology represented small oxide grains without cracks, pores, and blisters, as shown in Fig. 14a. SEM cross-sections showed that the oxide layer was regular and well-adhered, with an average thickness of 4 µm. Na was presented on the upper corrosion scale with a thickness of 0.5 µm, the Al layer was below the Na layer with a thickness of 0.8 µm, and the main layer with a composition of O and Ti had a thickness of 4 µm, as shown in Fig. 14 c. The morphology of O800-50-NaCl was analyzed by EDS, where point 1 was composed of Ti, O, Al, and tiny concentrations of Na and Cl, as presented in Table 3. Point 2 was composed of Ti, O, and Al, where Al had a high surface concentration, as shown in Table 3.

**Figure 15:**
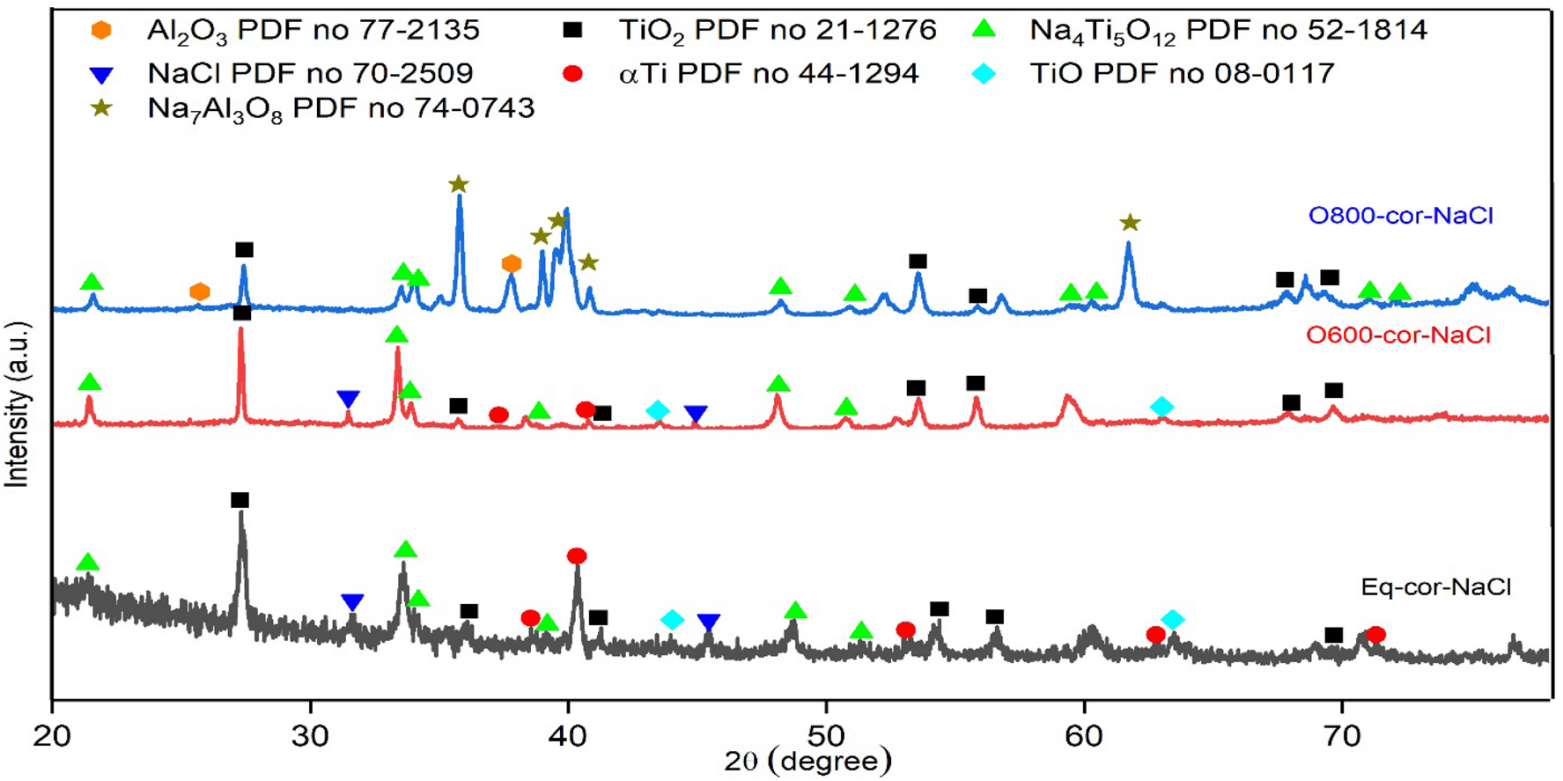
XRD patterns of NaCl corrosion raw O600 and O800 samples at 600 °C for 5 cycles. *Reference codes: Na_4_Ti_5_O_12_ 52-1814, NaCl 70-2509, TiO 08-0117 and Na_7_Al_3_O_8_ 74-0743.

Figure 15 shows the XRD patterns of raw, O600-50, and O800-50 samples after NaCl hot corrosion at 600 °C for 5 cycles. TiO, NaCl, α-Ti, Al_2_O_3_, TiO_2_, and Na_4_Ti_5_O_12_ phases were formed as the corrosion products. The raw samples involved NaCl, α-Ti, TiO_2_, and Na_4_Ti_5_O_12_ phases, where the corrosion phases were major and protective phases were minor based on the concentration of the phase on the corrosion surface. Those phases appeared for corroded O600 samples. The protective phases (α-Ti, Al_2_O_3_, and TiO_2_) were dominant in corroded O800 samples. The O600 samples were highly damaged after NaCl hot corrosion. In the case of O600-5, the weight loss was nearly equal to those of the raw samples after hot corrosion and then reduced to half that of O600-50 but remained unprotective. O600-50 had a very thin protective layer of TiO_2_ and did not form the protected Al_2_O_3_. The O800 samples were highly protective after NaCl hot corrosion, where the weight gain was observed at O800-5 and O800-20, but the weight change of O800-50 was a loss. The O800 samples had thick TiO_2_ and thin Al_2_O_3_ layers, enhancing their corrosion resistance. A weight loss study of O800-20 and O800-50 samples in which the oxide layer separated due to stress caused by a thermal expansion difference between the oxide layer and the metal substrate. Al_2_O_3_ had good corrosion resistance, and its consumption rate was very low at 600 ℃. The results showed that the O600 samples showed enhanced corrosion resistance with the increased oxidation time after NaCl hot corrosion. The increased oxidation temperature enhanced corrosion resistance (O800 samples) but decreased with the extended oxidation time.

**Figure 16:**
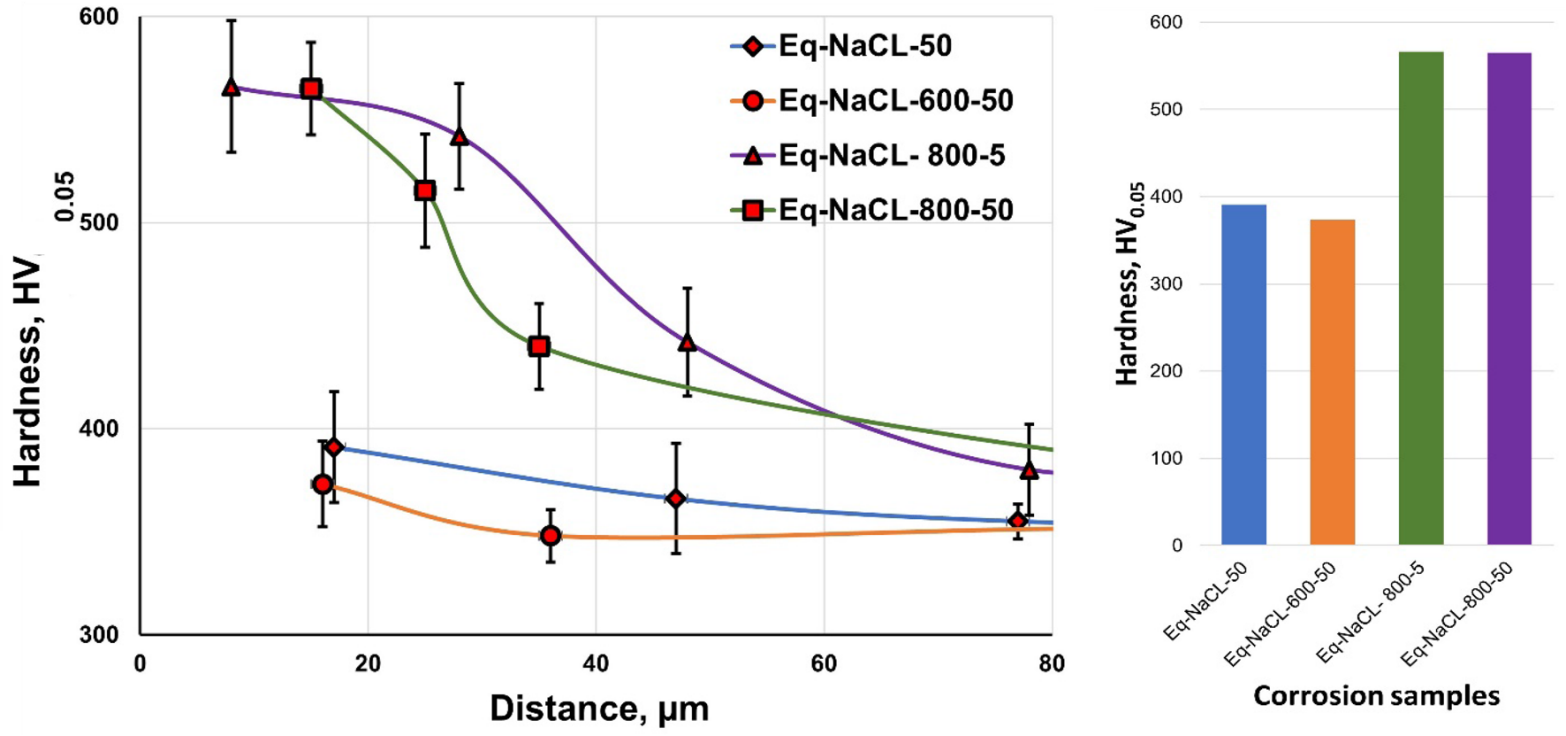
Relation between microhardness for raw O600 and O800 samples and depth after NaCl hot corrosion at 600 °C for 5 cycles.

Figure 16 shows microhardness measurements with depth after NaCl hot corrosion at 600 °C for 5 cycles. The microhardness was represented vis-à-vis the depth of the hardened region due to the dissolution of oxygen, where the hardened region was highly brittle and caused crack initiation. The raw and O600 samples after NaCl hot corrosion showed hardness change with the depth of the hardened region. The microhardness of raw samples was higher than those of O600 samples after NaCl hot corrosion, and the microhardness of O600 samples decreased faster than those of the raw samples. The raw samples showed more oxygen dissolution and more depth was hardened. The microhardness of O800 samples was higher on the surface after NaCl hot corrosion since the oxide layer was protective and oxygen dissolution reduced and stopped with the increased depth.

Ti and NaCl react above 400 °C, where the NaCl corrosion process reduces corrosion protection^53,54^. According to many researchers, the standard Gibbs free energy is negative for NaCl reactions at 600 °C, so these reactions are thermodynamically spontaneous. The NaCl corrosion process started when the deposited NaCl and the neutral-formed TiO_2_ reacted at 600 °C, producing Na_4_Ti_5_O_12_ and Cl_2_. XRD and EDS element map results showed the presence of Na_4_Ti_5_O_12_ on the top surface and that Cl diffused through cracks and pores toward the metal–oxide interface, where Ti reacted with Cl to form TiCl_4_. TiCl_4_ is volatile and thus can be transferred to the surface through cracks, where the partial pressure P(O_2_) increase leads to the reaction between TiCl_4_ and O_2_, forming TiO_2_ and Cl. The formed TiO_2_ was porous and nonprotective, whereas Cl_2_ was volatile, resulting in cycling corrosion reactions on the metal–oxide interface. A layer of Al_2_O_3_ was detected, and as a result, NaCl corrosion was less affected, where Al_2_O_3_ was continuous and did not allow the penetration of corrosive elements^27,55^. The results after examination showed that titanium alloys require protection against hot corrosion. The oxidation process formed suitable protective oxide layers at 800 °C, which can protect titanium alloy components against hot corrosion.

### NaCl + Na_2_SO_4_ hot corrosion

**Figure 17:**
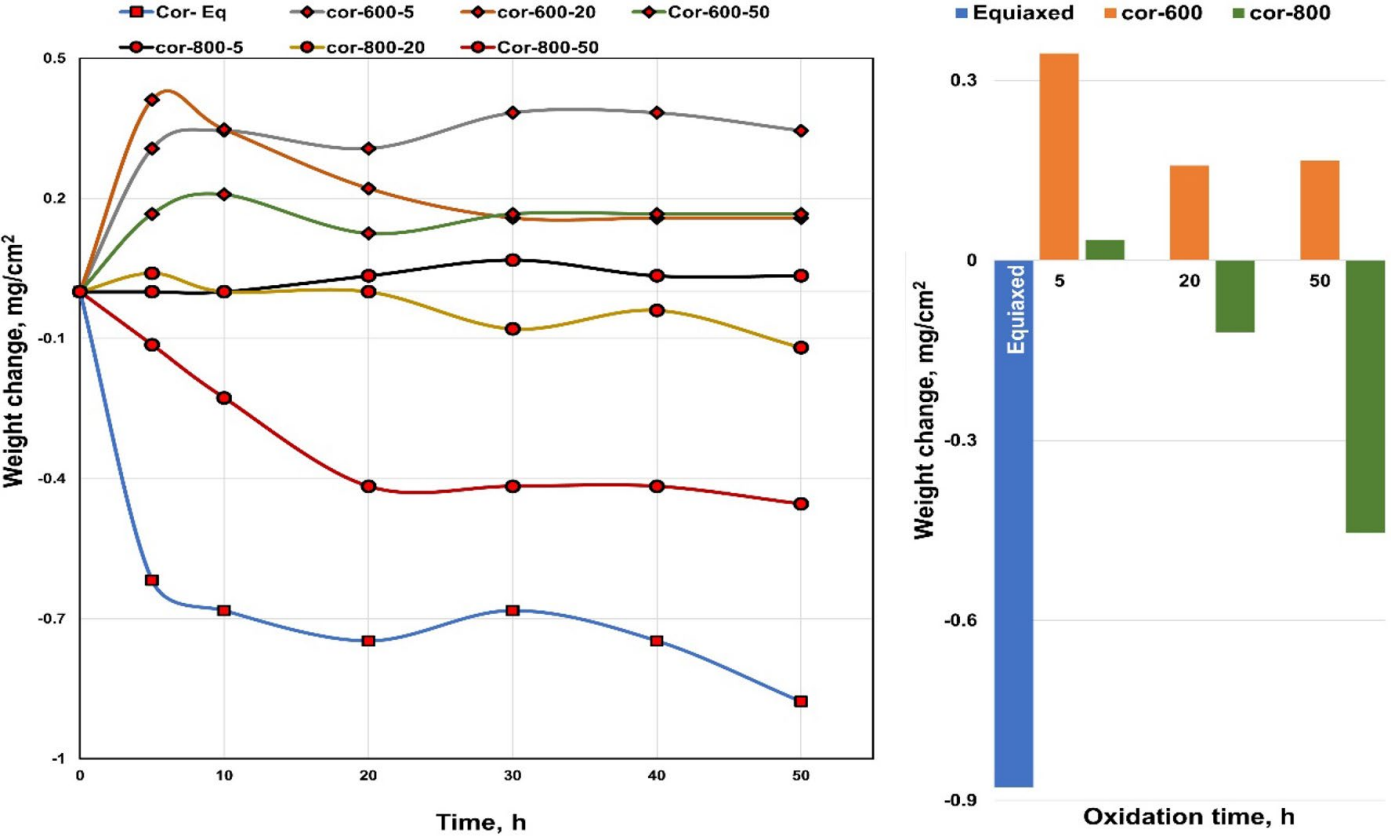
Weight change of raw, O600 and O800 samples as a function of time hot corrosion mixture NaCl + Na_2_SO_4_ at 600 °C for 5 cycles.

**Figure 18:**
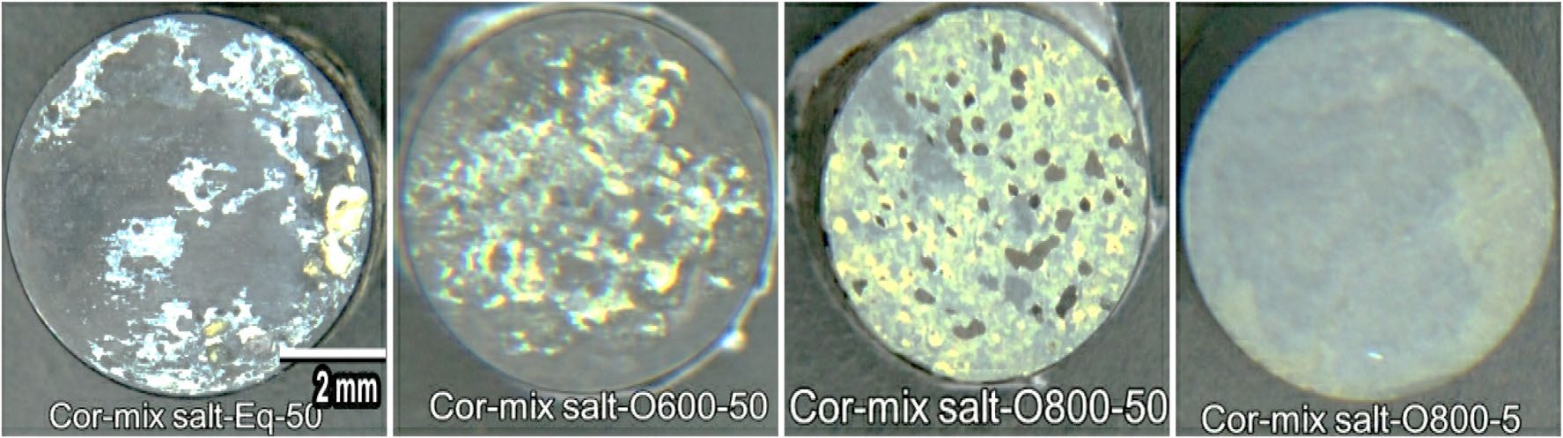
Surface morphology of raw, O600-50, O800-5, and O800-50 under hot corrosion mixture NaCl + Na_2_SO_4_ at 600 °C for 50 h.

Figure 17 shows the weight changes in raw O600 and O800 samples as a function of time upon NaCl + Na_2_SO_4_ hot corrosion at 600 °C with 10-h steps for 50 h shown in line and bar charts. NaCl + Na_2_SO_4_ hot corrosion caused less damage on the alloy surface than NaCl hot corrosion. Figure 18 shows the surface morphology of the raw, O600-50, O800-50, and O800-5 samples after the NaCl + Na_2_SO_4_ hot corrosion cycle at 600 °C for 5 cycles. For raw samples, the appearance of the metallic-white color changed to black and yellow without any deformation in shape after corrosion. The weight dropped after 5 h, and the weight change between 10 and 50 h was slow, showing a weight loss of 0.9 mg/cm^2^. The O600 samples changed the blue color to black and yellow without spallation after corrosion. The weight gain was observed for all O600 samples, where it was 0.34 mg/cm^2^ for O600-5 and 0.16 mg/cm^2^ for each O600-20 and O600-50. For the O800 samples, the appearance was unchanged, and spallation was observed for O800-50 after corrosion. The weight gain of O800-5 was 0.03 mg/cm^2^, and the weight losses of O800-20 and O800-50 were 0.12 and 0.4 mg/cm^2^, respectively. The raw samples experienced weight loss due to spallation, whereas the O600 samples formed oxide layers to protect against corrosion. O800-5 had good corrosion resistance, but O800-20 and O800-50 showed weak corrosion resistance because of spallation.

**Figure 19:**
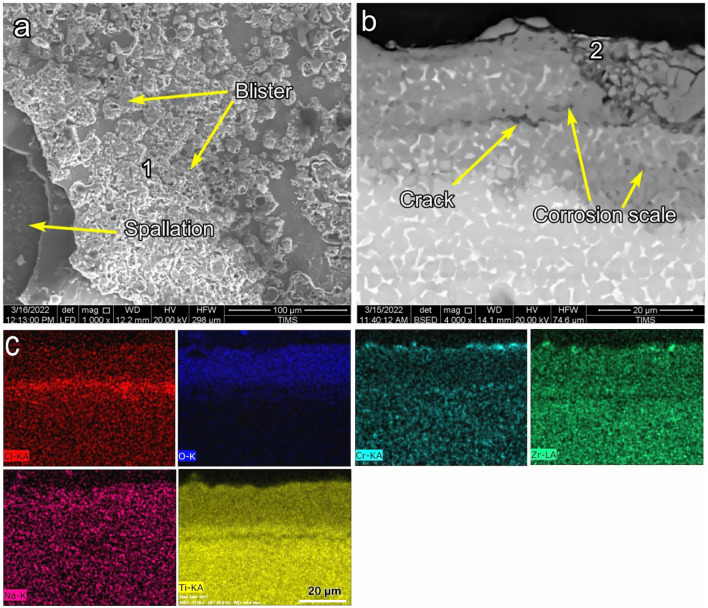
SEM and EDS element map concentration of the raw samples after NaCl + Na_2_SO_4_ hot corrosion at 600 °C for 5 cycles.

Figure 19 shows the SEM images of the surface morphology and cross-section and EDS elemental maps of raw samples after NaCl + Na_2_SO_4_ hot corrosion (raw-NaCl + Na_2_SO_4_) at 600 °C for 5 cycles. The raw sample showed blisters and the spallation of corrosion scales, as illustrated in Fig. 19a. The thicknesses of the corrosion scales were 20 μm, where the adhesion was weak between the scale and the base metal. In some regions, the material spalled off; in others, cracks occurred between the base metal and the corrosion scale, as shown in Fig. 19b. The EDS map showed high O, Ti, Cl, Cr, Zr, and Na concentrations throughout the corrosion scale (Fig. 19c). Cr and Zr were discontinuously distributed on the top surface of the corrosion scale. O was shown in the scale with a thickness of 17 µm. Cl was shown between the scale and the base metal in the region with a thickness of 4 µm. S was also presented on the surface of the corrosion scale at point 1, as shown in Table 2. The cross-sections of raw-NaCl + Na_2_SO_4_ samples were analyzed by EDS, and Ti, O, Al, and an alloying element were detected at point 2, as shown in Table 4.

**Table 4:** EDS point analysis (at.%) for raw and O600-50 and O800-50 morphology and cress section samples after NaCl + Na_2_SO_4_ hot corrosion at 600 °C.

Samples	Point	O	Na	Al	S	Ti	Cr	Zr	Nb	Mo	Sn
Raw + NaCl + Na_2_SO_4_	1	67.6	3.6	1.1	0.2	27	–	–	–	0.3	0.2
	2	64.3	–	2.4	–	30.3	1.7	0.7	–	0.3	0.3
O600-50 + NaCl + Na_2_SO_4_	1	80.3	0.8	1.8	0.1	16.9	–	–	–	0.1	–
	2	28.3	–	7.7	–	62.1	0.7	0.1	0.2	0.5	0.4
O800-50 + NaCl + Na_2_SO_4_	1	75	1.6	4.1	0.1	17.1	–	–	Cl–1.3	0.3	0.5
	2	75.2	0.3	11.6	–	12.6	0.1	0.1	0.1	–	–

**Figure 20:**
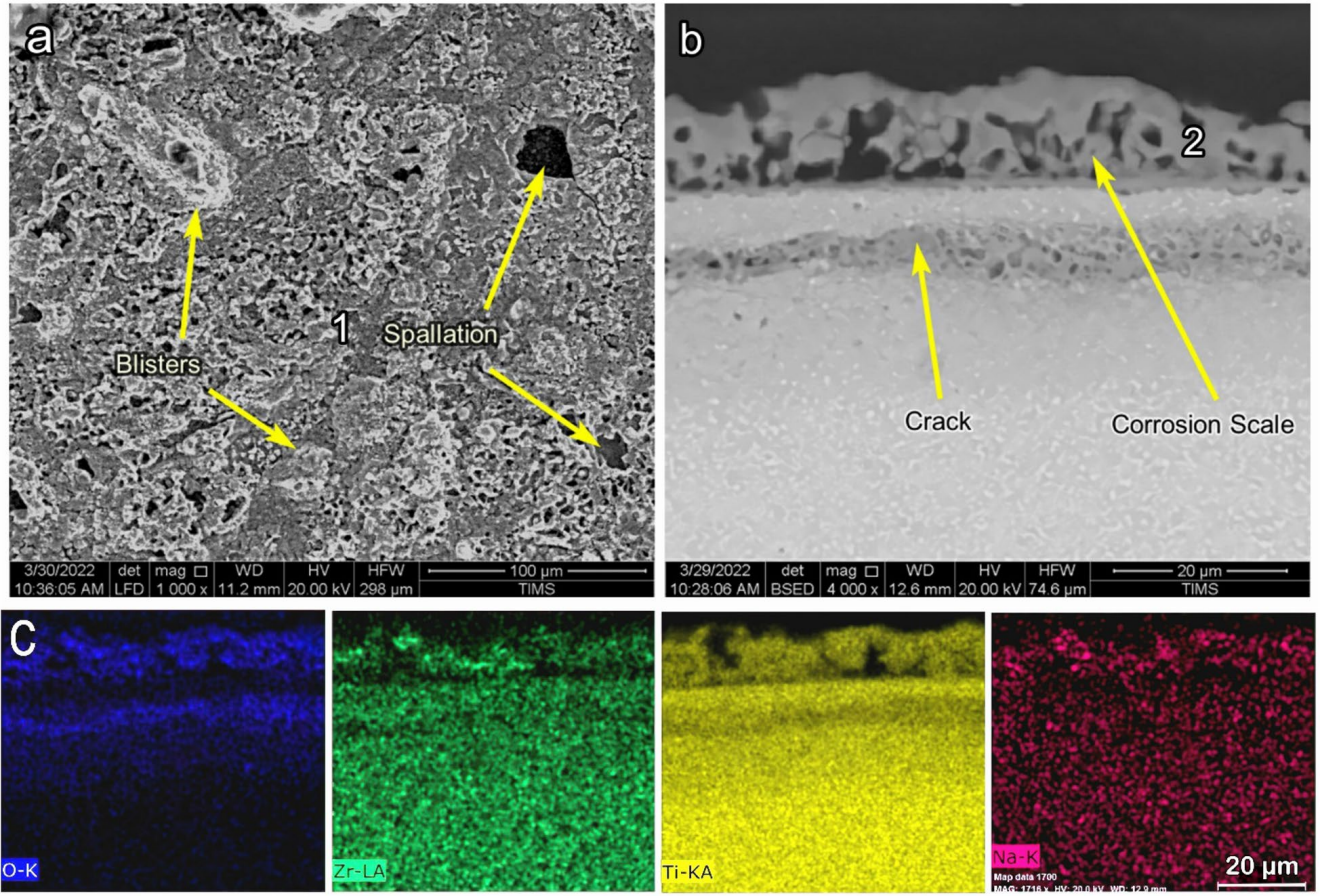
SEM and EDS element map concentration of O600-50 samples after NaCl + Na_2_SO_4_ hot corrosion at 600 °C during 5 cycles.

Figure 20 shows the SEM images of the surface morphology and cross-section and EDS elemental maps of O600-50 samples after NaCl + Na_2_SO_4_ hot corrosion (O600-50-NaCl + Na_2_SO_4_) at 600 °C for 5 cycles. The O600-50 samples showed blisters, cracks, and a tiny spallation region of corrosion scales, as illustrated in Fig. 20a. The corrosion scales had a 12 μm thickness, whereas the scales had pores and weak adhesion. A crack was shown in the base metal with a depth of 17 μm from the surface, as shown in Fig. 20b. The EDS maps showed the elements affected in the corrosion scale as O, Ti, Zr, and Na (Fig. 20c). Zr and Na showed high concentrations at the top surface of the scale. O was shown in the scale and cracks. S and Na had tiny concentrations at point 1, as shown in Table 4. The cross-sections of O600-50-NaCl + Na_2_SO_4_ samples analyzed by EDS showed Ti, O, and an increased concentration of Al (at point 2), as shown in Table 3.

**Figure 21:**
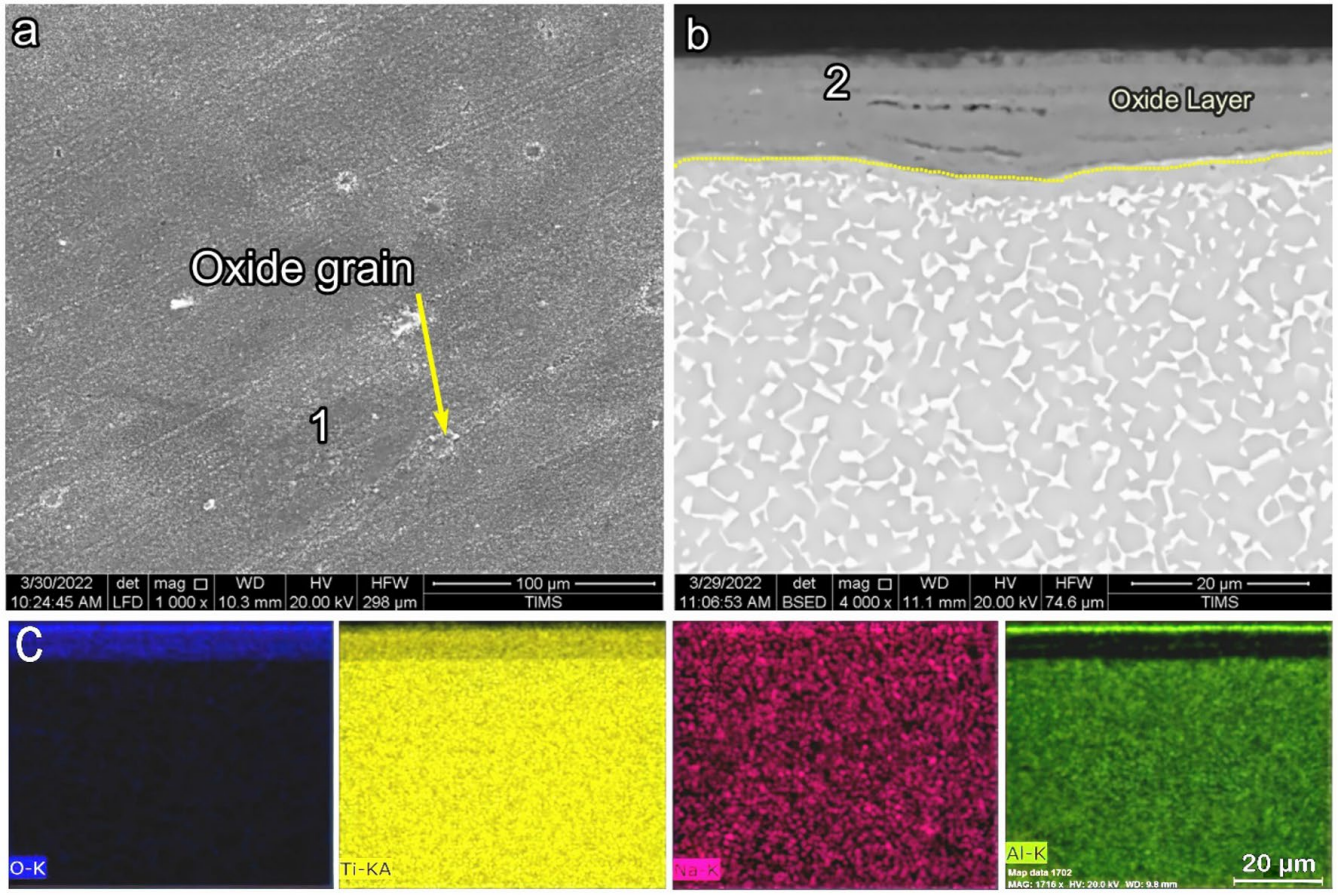
SEM and EDS element map concentration of O800-50 samples after NaCl + Na_2_SO_4_ hot corrosion at 600 °C during 5 cycles.

Figure 21 shows the SEM images of the surface morphology cross-sections and EDS elemental maps of O800-50 samples after NaCl + Na_2_SO_4_ hot corrosion at 600 °C for 5 cycles. The O800-50 samples showed oxide grains free of blisters, cracks, and spallation, as illustrated in Fig. 21a. The oxide layer was continuous with a thickness of 7 μm, as shown in Fig. 21b. The EDS maps showed the elements affected in the oxide layer as O, Ti, Al, and Na, as shown in Fig. 21c. Na was not observed, and the O layer thickness was 7 μm, indicating a high concentration in the oxide layer. The Al layer was continuous, and the adhesion layer on top of the oxide layer had a thickness of 1.5 µm. S, Na, and Cl were present at point 1 since the salt was adhered, as shown in Table 4. EDS analyzed the cross-sections of O800-50-NaCl + Na2SO4 samples. Ti, O, and a high concentration of Al were detected at point 2 due to alumina layer formation, as shown in Table 3.

**Figure 22:**
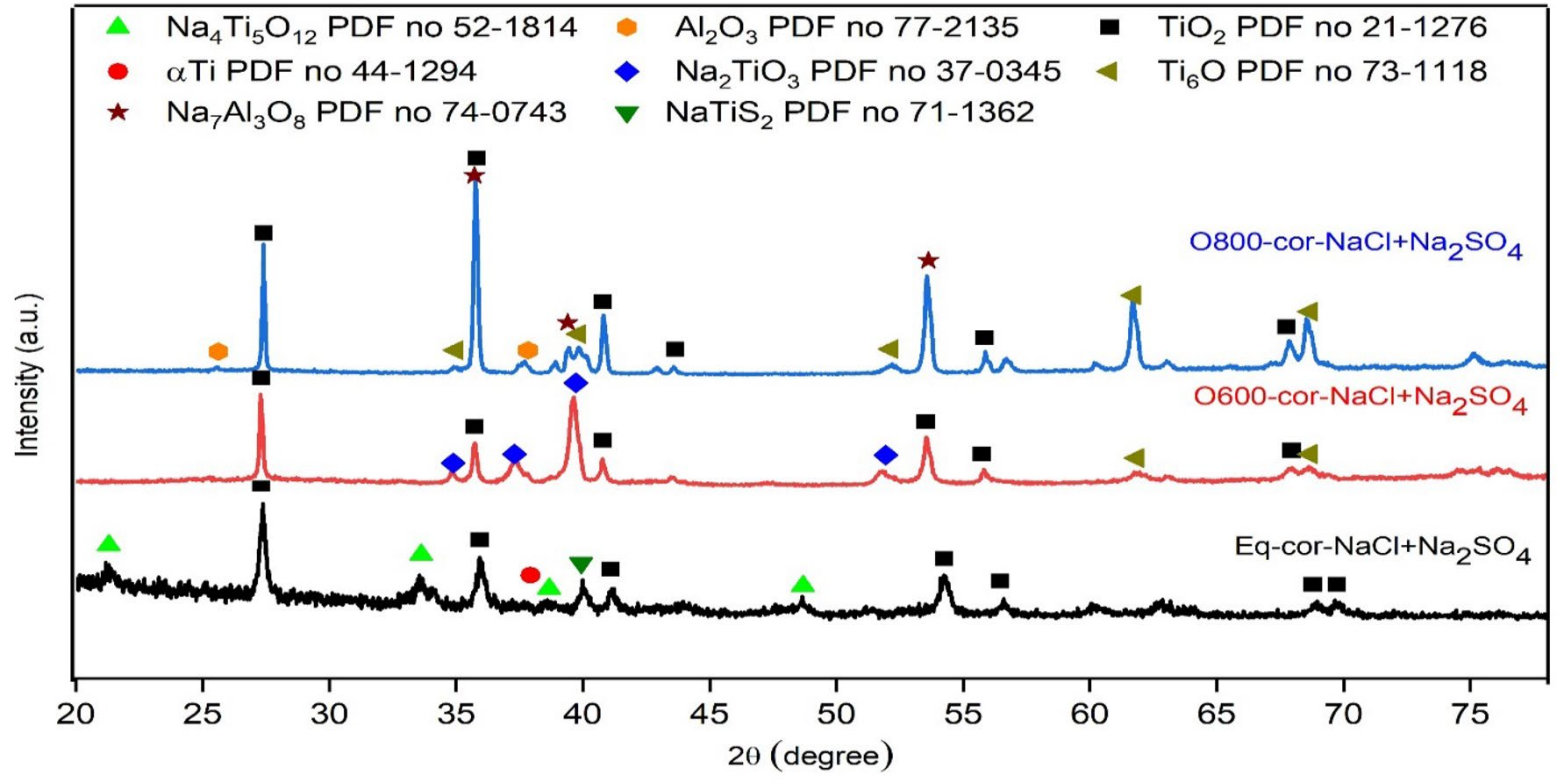
XRD patterns of NaCl + Na_2_SO_4_ corrosion raw O600 and O800 samples at 600 °C for 5 cycles. *Reference codes: Na_2_TiO_3_ 37-0345, Ti_6_Ol 73-1118, and Na_2_TiS_2_ 71-1362.

Figure 22 shows the XRD patterns of the raw, O600-50, and O800-50 samples after NaCl + Na_2_SO_4_ hot corrosion at 600 °C for 5 cycles. Ti_6_O, Na_2_TiO_3_, αTi, Al_2_O_3_, TiO_2_, and Na_4_Ti_5_O_12_ phases were found after the XRD investigation of the corrosion products. The raw samples involved αTi, TiO_2_, and Na_4_Ti_5_O_12_ phases. The O600 samples primarily involved Na_2_TiO_3_ and TiO_2_. The protective phases were presented as Ti_6_O, Al_2_O_3_, and TiO_2_ for corroded O800 samples.

**Figure 23:**
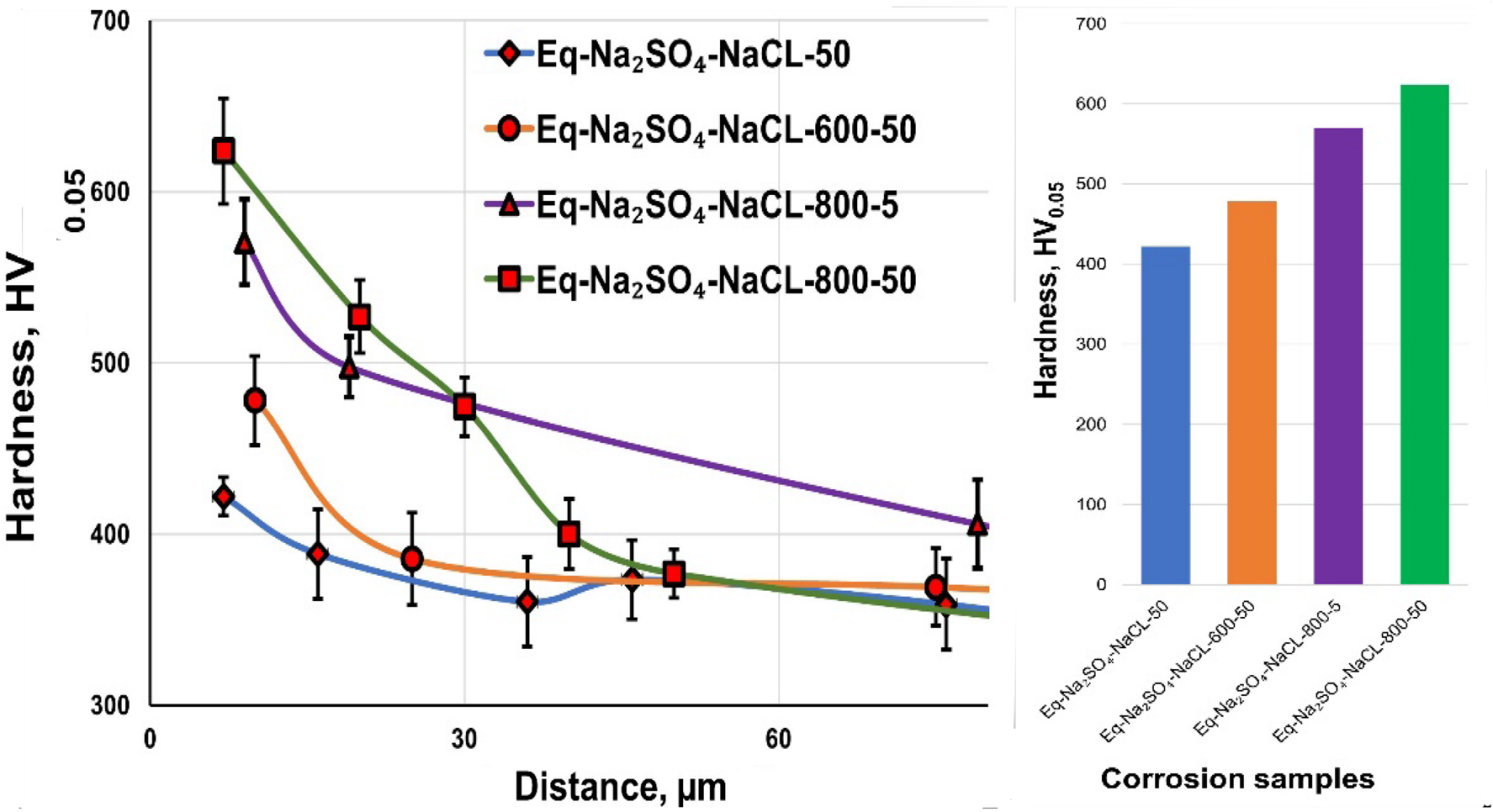
Relation between microhardness for raw O600 and O800 samples and depth after NaCl + Na_2_SO_4_ hot corrosion at 600 °C for 5 cycles.

Figure 23 shows the effects of NaCl + Na_2_SO_4_ hot corrosion on the microhardness values of the raw, O600-50, O800-5, and O800-50 samples. The microhardness versus the depth of the hardened samples was measured to determine the hardened, highly brittle layer thickness. The microhardness of raw samples was 422 HV_0.05_. The depth of raw samples hardened after NaCl + Na_2_SO_4_ hot corrosion was 30 μm. The O600-50 samples after NaCl + Na_2_SO_4_ hot corrosion showed a microhardness of 478 HV_0.05_ and a 20 μm hardened layer depth, similar to that under the same oxidation condition. The microhardness of O800 samples after NaCl + Na_2_SO_4_ hot corrosion was higher in the oxide layer and similar to that under the same oxidation condition.

The reduced damage of the mixture of Na_2_SO_4_ + NaCl salts was effective in Ti above 645 °C^53,56–58^. The presence of Na_2_SO_4_ formed internal sulfidation, which is detrimental due to the reduction of Cl^54,59,60^. The same reaction of NaCl active hot corrosion with Ti was used in the case of Na_2_SO_4_ + NaCl hot corrosion. Na_2_SO_4_ cracked to generate SO_3,_ which penetrated cracks or pores into the scale and then formed a metal sulfide layer. This layer acts as a protective barrier, preventing or slowing Cl_2_ diffusion to the metal. The oxide layer prevents the penetration of the corrosive element; thus, it must be deposited on the surface. Titanium alloys without protective oxide layers get damaged after hot corrosion and experience failure during service. The thermal oxidation process was used to form an oxide layer, which can protect the titanium alloy against Na_2_SO_4_ + NaCl hot corrosion.

## Conclusions

Thermal oxidation was used to form protective oxide layers, which enhanced the mechanical properties (hardness) and corrosion behavior of equiaxed TC21 Ti alloy. In addition, NaCl and Na_2_SO_4_ + NaCl hot corrosion were applied to the raw and oxidized samples. The following conclusions were obtained:

1. The oxide layer thickness (0.8, 2.4, 2.3, and 5.7 µm for O600-50, O700-50, O800-5, and O800-50, respectively) increased with oxidation temperature and time. The main oxide layer phases were TiO_2_ (rutile) and a small amount of Al_2_O_3_.
2. Thermal oxidation improved the hardness of raw samples from 342 ± 20 HV_0.05_ to 642 ± 36, 807 ± 63, 800 ± 20, and 900 ± 60 HV_0.05_ for O600-50, O700-50, O800-50, and O800-50, respectively.
3. The presence of solid NaCl hot corrosion deposits on the alloy surface caused catastrophic damage. NaCl hot corrosion was applied to oxide samples, where the weight losses of O600-50 and O800-50 were 5.5 and 1.5 mg/cm^2^, respectively. The weight loss of raw NaCl was 13.2 mg/cm^2^, and the samples had an irregular outer shape. The weight gain of O800-5 was 2.9 mg/cm^2^, where corrosion resistance was improved.
4. The presence of NaCl + Na_2_SO_4_ hot corrosion caused less damage on the alloy surface than NaCl hot corrosion. The weight loss of raw + NaCl + Na_2_SO_4_ was 0.9 mg/cm^2^. NaCl + Na_2_SO_4_ hot corrosion was applied to oxide samples, where the weight losses of O800-20 and O800-50 were 0.12 and 0.4 mg/cm^2^ due to thermal stress, and the weight gains of O600-50 and O800-5 were 0.34 and 0.03 mg/cm^2^, respectively. Meanwhile, the corrosion resistance of O800-5 remarkably improved.

## Data Availability

All data generated or analyzed during this study are included in this published article.

## References

[1] Loto RT (2022). Effect of heat-treatment processes and high temperature variation of acid-chloride media on the corrosion resistance of B265 (Ti–6Al–4V) titanium alloy in acid-chloride solution. Open Eng..

[2] Elshaer RN, Ibrahim KM (2022). Study of microstructure, mechanical properties, and corrosion behavior of as-cast Ni-Ti and Ti-6Al-4V alloys. J. Mater. Eng. Perform..

[3] Chen W (2022). Corrosion degradation behaviors of Ti6Al4V alloys in simulated marine environments. Coatings.

[4] Schutz, R. W. Defining the corrosion performance window for grade 28 titanium. In *CORROSION 2003* (OnePetro, 2003).

[5] Yaya K, Khelfaoui Y, Malki B, Kerkar M (2011). Numerical simulations study of the localized corrosion resistance of AISI 316L stainless steel and pure titanium in a simulated body fluid environment. Corros. Sci..

[6] Barril S, Mischler S, Landolt D (2004). Influence of fretting regimes on the tribocorrosion behaviour of Ti6Al4V in 0.9 wt.% sodium chloride solution. Wear.

[7] Narayanan R, Seshadri SK (2008). Point defect model and corrosion of anodic oxide coatings on Ti–6Al–4V. Corros. Sci..

[8] Tamilselvi S, Raman V, Rajendran N (2010). Evaluation of corrosion behavior of surface modified Ti–6Al–4V ELI alloy in hanks solution. J. Appl. Electrochem..

[9] Thomas R (2003). Titanium in the geothermal industry. Geothermics.

[10] Ibrahim KM, El-Hakeem AMM, Elshaer RN (2013). Microstructure and mechanical properties of cast and heat treated Ti–6.55 Al–3.41 Mo–1.77 Zr alloy. Trans. Nonferrous Met. Soc. China.

[11] Sun C, Xiao R, Li H, Ruan Y (2022). Effects of phase selection and microsegregation on corrosion behaviors of Ti-Al-Mo alloys. Corros. Sci..

[12] Bordbar-Khiabani A, Gasik M (2023). Electrochemical and biological characterization of Ti–Nb–Zr–Si alloy for orthopedic applications. Sci. Rep..

[13] Elshaer, R. N. Effect of initial α-phase morphology on microstructure, mechanical properties, and work-hardening instability during heat treatment of TC21 Ti-alloy. *Metallogr. Microstruct. Anal.* 1–16 (2022).

[14] Abdelmoneim A, Elshaer RN, El-Shennawy M, Sobh AS (2023). Modeling of wear resistance for TC21 Ti-alloy using response surface methodology. Sci. Rep..

[15] Yadav P, Saxena KK (2020). Effect of heat-treatment on microstructure and mechanical properties of Ti alloys: An overview. Mater. Today Proc..

[16] Elshaer, R. N., Abdelhameed, M., Ibrahim, K. M., El-Shennawy, M. & Sobh, A. Static and fatigue characteristics of heat-treated Ti–6Al–3Mo–2Zr–2Sn–2Nb–1.5 Cr–0.1 Si alloy. *Metallogr. Microstruct. Anal.* 1–11 (2022).

[17] Elshaer RN, El-Deeb MSS, Mohamed SS, Ibrahim KM (2022). Effect of strain hardening and aging processes on microstructure evolution, tensile and fatigue properties of cast Ti-6Al-2Sn-2Zr–2Mo-1.5 Cr-2Nb-0.1 Si alloy. Int. J. Met..

[18] Sharifi F (2019). The effect of different heat treatment cycle on hot corrosion and oxidation behavior of Ti–6Al–4V. Mater. Res. Express.

[19] Guleryuz H, Cimenoglu H (2009). Oxidation of Ti-6Al-4V alloy. J. Alloys Compd..

[20] Poquillon D, Armand C, Huez J (2013). Oxidation and oxygen diffusion in Ti–6al–4V alloy: Improving measurements during sims analysis by rotating the sample. Oxid. Met..

[21] Kumar S, Chattopadhyay K, Mahobia GS, Singh V (2016). Hot corrosion behaviour of Ti–6Al–4V modified by ultrasonic shot peening. Mater. Des..

[22] Çomaklı O, Yazıcı M, Yetim T, Yetim AF, Çelik A (2018). Effect of Ti amount on wear and corrosion properties of Ti-doped Al_2_O_3_ nanocomposite ceramic coated CP titanium implant material. Ceram. Int..

[23] Krawiec H, Vignal V, Loch J, Erazmus-Vignal P (2015). Influence of plastic deformation on the microstructure and corrosion behaviour of Ti–10Mo–4Zr and Ti–6Al–4V alloys in the Ringer’s solution at 37 C. Corros. Sci..

[24] Ahmed FS, El-Zomor MA, Ghazala MSA, Elshaer RN (2022). Effect of oxide layers formed by thermal oxidation on mechanical properties and NaCl-induced hot corrosion behavior of TC21 Ti-alloy. Sci. Rep..

[25] Loto, C. A., Loto, R. T. & Popoola, A. P. I. Synergistic effect of tobacco and kola tree extracts on the corrosion inhibition of mild steel in acid chloride (2011).

[26] Loto CA, Joseph OO, Loto RT (2014). Adsorption and inhibitive properties of *Camellia sinensis* for aluminium alloy in HCl. Int. J. Electrochem. Sci.

[27] Ahmed, F. S., El-Zomor, M. A., Abo Ghazala, M. S. & Elshaer, R. N. Influence of α-phase morphology on mechanical characteristics, cycle oxidation, and hot corrosion behavior of Ti-6Al-3Mo-2Nb-2Zr-2Sn-1.5 Cr alloy. *Metallogr. Microstruct. Anal.* 1–15 (2022).

[28] Ciszak C (2020). Degradation mechanism of Ti-6Al-2Sn-4Zr-2Mo-Si alloy exposed to solid NaCl deposit at high temperature. Corros. Sci..

[29] Zhang, M. *et al.* Corrosion behaviors of nitride coatings on titanium alloy in NaCl-induced hot corrosion. *Acta Metall. Sin. (English Letters)* 1–13 (2021).

[30] Anuwar M, Jayaganthan R, Tewari VK, Arivazhagan N (2007). A study on the hot corrosion behavior of Ti–6Al–4V alloy. Mater. Lett..

[31] Li R, Liu L, Cui Y, Liu R, Wang F (2022). Corrosion behavior of pure Ti under continuous NaCl solution spraying at 600 °C. npj Mater. Degrad..

[32] Shi H (2022). Effect of plasma electrolytic oxidation on the hot salt corrosion fatigue behavior of the TC17 titanium alloy. Mater. Corros..

[33] Béguin JD (2008). Evaluation of Al_2_O_3_ MOCVD coating for titanium alloys protection under severe conditions at high temperature. Materials Science Forum.

[34] Xiong Y, Guan C, Zhu S, Wang F (2006). Effect of enamel coating on oxidation and hot corrosion behaviors of Ti-24Al-14Nb-3V alloy. J. Mater. Eng. Perform..

[35] Chou K, Chu P-W, Marquis EA (2018). Early oxidation behavior of Si-coated titanium. Corros. Sci..

[36] Aniołek K, Kupka M, Barylski A (2019). Characteristics of the tribological properties of oxide layers obtained via thermal oxidation on titanium Grade 2. Proc. Inst. Mech. Eng. Part J J. Eng. Tribol..

[37] Ding Z-Y (2019). Influence of Al_2_O_3_ addition in NaAlO_2_ electrolyte on microstructure and high-temperature properties of plasma electrolytic oxidation ceramic coatings on Ti_2_AlNb alloy. Surf. Coat. Technol..

[38] Zhang L (2020). Oxidation resistance of plasma-sprayed double-layered LC/YSZ coatings with different thickness ratios at high temperatures. Oxid. Met..

[39] Elshaer RN, Elshazli AM, Hussein AHA, Al-Sayed SR (2022). Impact of laser process parameters in direct energy deposition on microstructure, layer characteristics, and microhardness of TC21 alloy. Int. J. Adv. Manuf. Technol..

[40] Elshazli AM, Elshaer RN, Hussein AHA, Al-Sayed SR (2021). Laser surface modification of TC21 (α/β) titanium alloy using a direct energy deposition (DED) process. Micromachines.

[41] Omoniyi PO, Akinlabi ET, Mahamood RM, Jen TC (2021). Corrosion resistance of heat treated Ti6Al4V in NaCl. Chem. Data Collect..

[42] Bower K, Murray S, Reinhart A, Nieto A (2020). Corrosion resistance of selective laser melted Ti–6Al–4V alloy in salt fog environment. Res. Mater..

[43] Chellaganesh D, Khan MA, Jappes JTW (2020). Hot corrosion behaviour of nickel–iron-based superalloy in gas turbine application. Int. J. Ambient Energy.

[44] Dai J (2021). High temperature oxidation and hot corrosion behaviors of Ti_2_AlNb alloy at 923 K and 1023 K. Corros. Sci..

[45] Casadebaigt A, Hugues J, Monceau D (2020). High temperature oxidation and embrittlement at 500–600 °C of Ti-6Al-4V alloy fabricated by Laser and Electron Beam Melting. Corros. Sci..

[46] Gaddam R, Sefer B, Pederson R, Antti M-L (2015). Oxidation and alpha-case formation in Ti–6Al–2Sn–4Zr–2Mo alloy. Mater. Charact..

[47] Kumar A, Kushwaha MK, Israr M, Kumar R (2020). Evaluation of mechanical properties of titanium alloy after thermal oxidation process. Trans. Indian Inst. Met..

[48] Aniołek K (2020). Structure and properties of titanium and the Ti-6Al-7Nb alloy after isothermal oxidation. Surf. Eng..

[49] Liu, H., Hao, S., Wang, X. & Feng, Z. Interaction of a near-{alpha} type titanium alloy with NiCrAlY protective coating at high temperatures. *Scr. Mater.* **39** (1998).

[50] Aniołek K, Barylski A, Kupka M, Dercz G (2020). Cyclic oxidation of titanium grade 2. Mater..

[51] Wang S, Liang Y, Sun H, Feng X, Huang C (2021). oxygen induced phase transformation in TC21 alloy with a lamellar microstructure. Metals.

[52] Aniołek K, Kupka M (2019). Mechanical, tribological and adhesive properties of oxide layers obtained on the surface of the Ti–6Al–7Nb alloy in the thermal oxidation process. Wear.

[53] Fan L (2019). Effect of streaming water vapor on the corrosion behavior of Ti60 alloy under a solid NaCl deposit in water vapor at 600 °C. Corros. Sci..

[54] Ciszak C, Popa I, Brossard J-M, Monceau D, Chevalier S (2016). NaCl induced corrosion of Ti-6Al-4V alloy at high temperature. Corros. Sci..

[55] Xiong Y, Zhu S, Wang F (2008). Synergistic corrosion behavior of coated Ti60 alloys with NaCl deposit in moist air at elevated temperature. Corros. Sci..

[56] Zhang P, Li X-H, Moverare J, Peng RL (2020). The iron effect on hot corrosion behaviour of MCrAlX coating in the presence of NaCl at 900 °C. J. Alloys Compd..

[57] Jiang CY (2018). A Zr-doped single-phase Pt-modified aluminide coating and the enhanced hot corrosion resistance. Corros. Sci..

[58] Fan L (2016). Corrosion behavior of Ti60 alloy under a solid NaCl deposit in wet oxygen flow at 600 C. Sci. Rep..

[59] Li R (2020). A new insight into the NaCl-induced hot corrosion mechanism of TiN coatings at 500 °C. Corros. Sci..

[60] Dai J, Zhu J, Chen C, Weng F (2016). High temperature oxidation behavior and research status of modifications on improving high temperature oxidation resistance of titanium alloys and titanium aluminides: A review. J. Alloys Compd..

